# Enhanced Glycolysis‐Driven Histone H3K18 Lactylation Regulates Epileptogenesis by Modulating the E3 Ubiquitin Ligase COP1

**DOI:** 10.1002/advs.202516985

**Published:** 2026-05-29

**Authors:** Yuan Meng, Yanlin Guo, Liumi Jiang, Jing Luo, Yan Liu, Ziwei Yuan, Pingyang Ke, Ran Duan, Fei Xiao

**Affiliations:** ^1^ Department of Neurology Chongqing Key Laboratory of Major Neurological and Mental Disorders Neurology Key Laboratory of Chongqing Education Commission of China Chongqing Key Laboratory of Neurology The First Affiliated Hospital of Chongqing Medical University Chongqing China; ^2^ Department of Rare Disease The First Affiliated Hospital of Chongqing Medical University Chongqing China

**Keywords:** COP1, epilepsy, excitation‐inhibition balance, histone lactylation, ubiquitination

## Abstract

Metabolic reprogramming is increasingly implicated in epilepsy, yet the mechanisms linking metabolic shifts to neuronal hyperexcitability remain elusive. Here, we identify lactate‐driven histone H3 lysine 18 lactylation (H3K18la), a novel post‐translational modification, as a critical epigenetic regulator of seizure susceptibility. Using kainic acid (KA)‐induced epilepsy models, we demonstrate that enhanced glycolytic flux through pyruvate kinase M2 (PKM2) generates excess lactate, driving robust H3K18la elevation in neurons during acute epileptogenesis. Chromatin immunoprecipitation sequencing (ChIP‐seq) analysis revealed that this epigenetic mark promotes transcriptional upregulation of the E3 ubiquitin ligase *Cop1*. COP1 subsequently promotes K48‐linked polyubiquitination and proteasomal degradation of the γ‐aminobutyric acid type A (GABA_A_) receptor β2 subunit (GABA_A_Rβ2), reducing inhibitory synaptic transmission, and heightening seizure susceptibility. Pharmacological or genetic targeting of PKM2 or genetic knockout of *Cop1* reversed these effects and conferred robust seizure protection. Our findings reveal a pathogenic metabolic‐epigenetic‐proteostatic pathway in epilepsy, offering new therapeutic targets for restoring brain metabolic and electrical homeostasis.

## Graphical abstract

Neuronal PKM2‐driven glycolysis generates excess lactate that triggers histone H3K18 lactylation (H3K18la), establishing a pathogenic metabolic‐epigenetic axis in epilepsy. Elevated H3K18la enriches the *Cop1* promoter, transcriptionally upregulating the E3 ubiquitin ligase COP1, which subsequently drives proteasomal degradation of GABA_A_Rβ2 and impairs inhibitory synaptic transmission. Pharmacological or genetic targeting of PKM2 or COP1 disrupts this cascade, conferring robust seizure protection, and revealing new therapeutic targets.

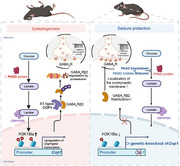

## 1 Introduction

Epilepsy affects approximately 70 million individuals worldwide, making it one of the most prevalent neurological disorders characterized by recurrent, unprovoked seizures arising from diverse etiologies including genetic mutations, brain trauma, infections, and developmental abnormalities [1, 2]. The pathophysiology of epilepsy involves complex disruptions in normal brain function, encompassing dysfunctional ion channels, imbalanced synaptic excitation and inhibition, and neuroinflammation cascades [3, 4, 5]. Despite significant advances in understanding these classical mechanisms, approximately 30% of patients develop drug‐resistant epilepsy that remains refractory to current antiepileptic drugs [6, 7]. This large treatment gap underscores an urgent need to identify novel pathogenic mechanisms and develop therapeutic strategies that extend beyond conventional targets like ion channels and neurotransmitter systems.

Emerging evidence has identified metabolic reprogramming as a fundamental feature of the epileptic brain, though the underlying mechanisms remain incompletely understood [8, 9, 10]. The brain has an exceptionally high energy demand that is acutely exacerbated during seizure activity, suggesting that neurons must adapt their metabolic processes to cope with this bioenergetic stress [8]. Clinical and preclinical studies consistently demonstrate elevated lactate levels—the end product of glycolysis—in epileptic foci during and after seizures [11, 12, 13, 14]. However, the precise role of this lactate accumulation in the pathophysiology of epilepsy remains a significant and unresolved paradox. On one hand, lactate can exert neuroprotective and anti‐convulsant effects, in part by activating the Gi‐coupled hydroxycarboxylic acid receptor 1 (HCAR1, also known as GPR81), which dampens neuronal activity [15, 16]. On the other hand, lactate is increasingly viewed as a pro‐convulsant molecule. Seminal work has shown that pharmacological inhibition of lactate dehydrogenase (LDH), the enzyme that produces lactate from pyruvate, confers potent seizure protection, suggesting that lactate production itself contributes to hyperexcitability [14, 17]. This apparent contradiction suggests that the downstream consequences of lactate elevation are more complex than previously appreciated and that lactate may signal through alternative, maladaptive pathways that have yet to be defined.

Recent advances in metabolic epigenetics have identified histone lactylation as a post‐translational modification that drily couples cellular metabolic states with epigenetic regulation of gene expression [18]. Unlike traditional histone modifications that primarily depend on cofactors derived from one‐carbon metabolism or acetyl‐CoA, lactylation utilizes lactyl‐CoA as a substrate, creating a direct mechanistic bridge between glycolytic flux and chromatin remodeling [19, 20]. Emerging research has begun to elucidate the role of histone lactylation in the central nervous system, where it has been implicated in neuroinflammation, synaptic plasticity, and the pathogenesis of neurodegenerative diseases, including Alzheimer's disease and Parkinson's disease [21, 22, 23, 24]. These findings raise the intriguing possibility that lactate accumulation in epilepsy could drive a pathological gene expression program through epigenetic reprogramming.

The maintenance of protein homeostasis, or proteostasis, through the ubiquitin‐proteasome system (UPS) represents a fundamental requirement for proper synaptic function and neurotransmission [25]. Disruption of proteostatic mechanisms has been increasingly recognized as a core feature of various neurological disorders, including epilepsy, where altered protein degradation pathways contribute to synaptic dysfunction and network hyperexcitability [26, 27]. Among the numerous E3 ubiquitin ligases that orchestrate protein turnover, COP1 (Constitutive photomorphogenic protein 1, also known as RFWD2) stands out for its high neuronal abundance and emerging roles in dendritic spine development, synaptic transmission, and neurodevelopmental phenotypes [28, 29, 30, 31]. Nevertheless, how COP1 itself is regulated in the epileptic brain, and whether it interfaces with epigenetic cues, is unknown.

The integration of metabolic signaling with epigenetic regulation represents a particularly attractive mechanism for understanding how transient metabolic changes during seizures could produce lasting alterations in neuronal excitability. Here, we investigated whether lactate accumulation in epilepsy drives pathological gene expression through histone lactylation. By combining mouse models of epilepsy with multi‐omics, biochemical, and electrophysiological analyses, we identified a novel pathway linking enhanced glycolysis to reduced inhibitory neurotransmission and seizure susceptibility. Our work establishes the lactate‐H3K18la‐COP1‐GABA_A_Rβ2 axis as a central mechanism linking metabolic dysregulation to epileptogenesis, thereby revealing a new set of potential therapeutic targets.

## 2 Results

### 2.1 Lactate Accumulates in the Hippocampus and Histone Lactylation is Elevated During Epileptogenesis

**FIGURE 1:**
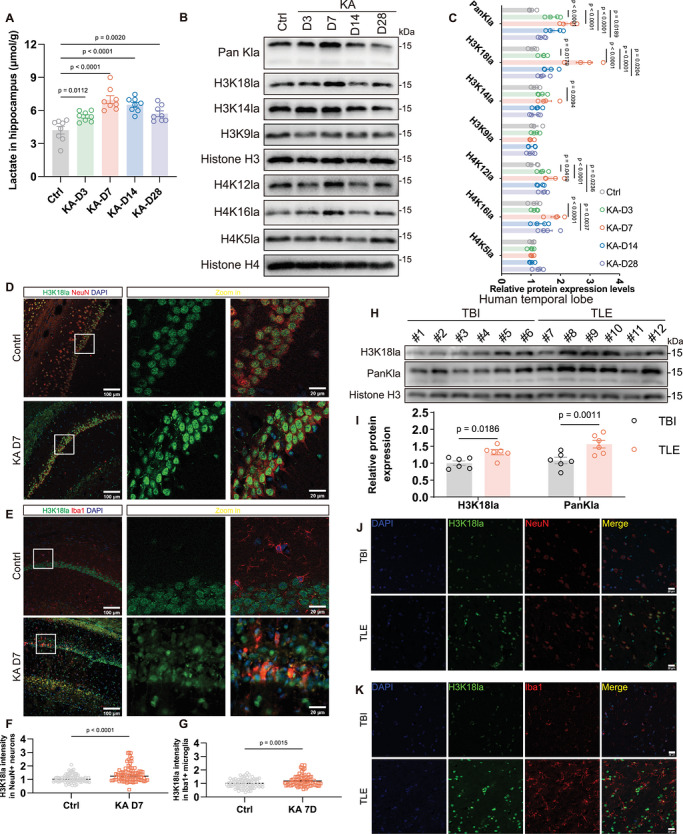
Lactate and histone lactylation are elevated in KA‐induced epileptic mice and human epileptic brain tissue. (A) Quantification of lactate levels in the hippocampus of control (Ctrl) mice and at 3, 7, 14, and 28 days post‐kainic acid (KA) injection (*n* = 8 mice per group). (B) Representative western blots of Panlysine lactylation (PanKla) and site‐specific histone lactylation marks (H3K18la, H3K14la, H3K9la, H4K12la, H4K16la, and H4K5la) from hippocampal tissue of Ctrl and KA‐treated mice at the indicated time points. Histone H3 and histone H4 were used as loading controls. (C) Densitometric quantification of relative protein expression of the histone lactylation marks shown in (B) (*n* = 4 mice per group). (D,E) Representative confocal microscopy images of the hippocampal CA1 region from Ctrl and KA‐D7 mice. Sections were co‐stained for H3K18la (green) and cell‐type‐specific markers (red): NeuN for neurons (D) and Iba1 for microglia (E). Nuclei were counterstained with DAPI (blue). Scale bars, 100 µm (main images), and 20 µm (insets). (F,G) Quantification of H3K18la fluorescence intensity in NeuN‐positive neurons (F) and Iba1‐positive microglia (G) (*n* = 3 mice, with 4–6 fields analyzed per mouse). (H) Representative western blots of H3K18la and PanKla from hippocampal tissue of human traumatic brain injury (TBI) controls and temporal lobe epilepsy (TLE) patients. Histone H3 was used as a loading control (*n* = 6 patients per group). (I) Densitometric quantification of H3K18la and PanKla levels shown in (H) (*n* = 6 patients per group). (J and K) Representative confocal microscopy images of hippocampal tissue from TBI and TLE patients. Sections were co‐stained for H3K18la (green) and NeuN (red) for neurons (J), or H3K18la (green), and Iba1 (red) for microglia (K). Nuclei were counterstained with DAPI (blue). Scale bars, 25 µm. Data are presented as mean ± SEM. Statistical significance was determined by one‐way ANOVA with Tukey's post hoc test (A and C) or an unpaired, two‐tailed Student's *t*‐test (F, G, and I). Exact p‐values are indicated in the figure. See also Figures S1 and S2.

Metabolic reprogramming has been increasingly recognized as a critical factor in neurological disorders, including epilepsy [32, 33]. Given that lactate serves as both a key energy currency and a signaling molecule in the brain [34], we first investigated its temporal dynamics in the hippocampus of mice following kainic acid (KA)‐induced status epilepticus, a well‐established model of temporal lobe epilepsy (TLE) [34]. Using a colorimetric assay, we found that hippocampal lactate levels were significantly elevated at 7 and 14 days post‐KA injection, peaking at day 7, before returning to near‐baseline levels by day 28 (Figure 1A). This temporal pattern suggests metabolic dysregulation during the acute epileptogenic phase. consistent with previous observations of altered brain energy metabolism following seizures [35].

Lactate is the precursor for histone lactylation, a recently discovered post‐translational modification that directly links metabolic status to epigenetic gene regulation [18]. We hypothesized that the seizure‐induced lactate elevation would alter histone lactylation patterns during epileptogenesis. Western blot analysis of acid‐extracted histones revealed dynamic changes across several lactylation sites (Figure 1B). Antibody specificity was confirmed by mass spectrometry, dot blot, and peptide competition assay, demonstrating selectivity for the lactylated but not the acetylated form of H3K18 (Figure S2). Notably, lysine 18 of histone H3 (H3K18la) was robustly and significantly increased, with a temporal profile that mirrored the changes in lactate, peaking at day 7 post‐KA injection (Figure 1C). Pan‐lysine lactylation (PanKla) was also elevated at day 7, while other marks such as H3K14la, H4K12la, and H4K5la showed more modest or transient changes. These results demonstrate that H3K18la is specifically upregulated during acute epileptogenesis. Similar findings were observed in a pentylenetetrazol (PTZ) kindling model, with H3K18la showing the largest increase among all tested sites (Figure S1D–F).

To identify the cell types of exhibiting elevated H3K18la, we performed immunofluorescence co‐staining with cell‐type‐specific markers. Co‐localization analysis with NeuN revealed significantly elevated H3K18la intensity in hippocampal neurons at 7 days post‐KA (Figure 1D,F). Similarly, co‐staining with Iba1 demonstrated increased H3K18la levels in activated microglia following KA treatment (Figure 1E,G). Astrocytic H3K18la levels remained unchanged in the epileptic hippocampus (Figure S1A–B). Together, these results demonstrate that seizure‐induced lactate accumulated in the hippocampus and that H3K18la modification is significantly increased, primarily within neurons and microglia, linking metabolic perturbation directly to epigenetic changes in the cells most critical to seizure pathology.

To determine whether H3K18la elevation extends to human epileptic brain tissue, we analyzed hippocampal specimens from TLE patients and traumatic brain injury (TBI) controls. Western blot analysis revealed significantly elevated levels of both H3K18la and PanKla in TLE tissue compared with TBI controls (Figure 1H,I). Immunofluorescence co‐staining further confirmed that H3K18la was markedly increased in NeuN‐positive neurons (Figure 1J) and Iba1‐positive microglia (Figure 1K) in TLE patients, while H3K18la signal in GFAP‐positive astrocytes was not elevated (Figure S1C). These findings establish H3K18la upregulation as a conserved feature of epileptic tissue in both rodent models and human disease.

### 2.2 Enhanced Glycolytic Flux Through PKM2 Drives H3K18la and Promotes Seizure Susceptibility

**FIGURE 2:**
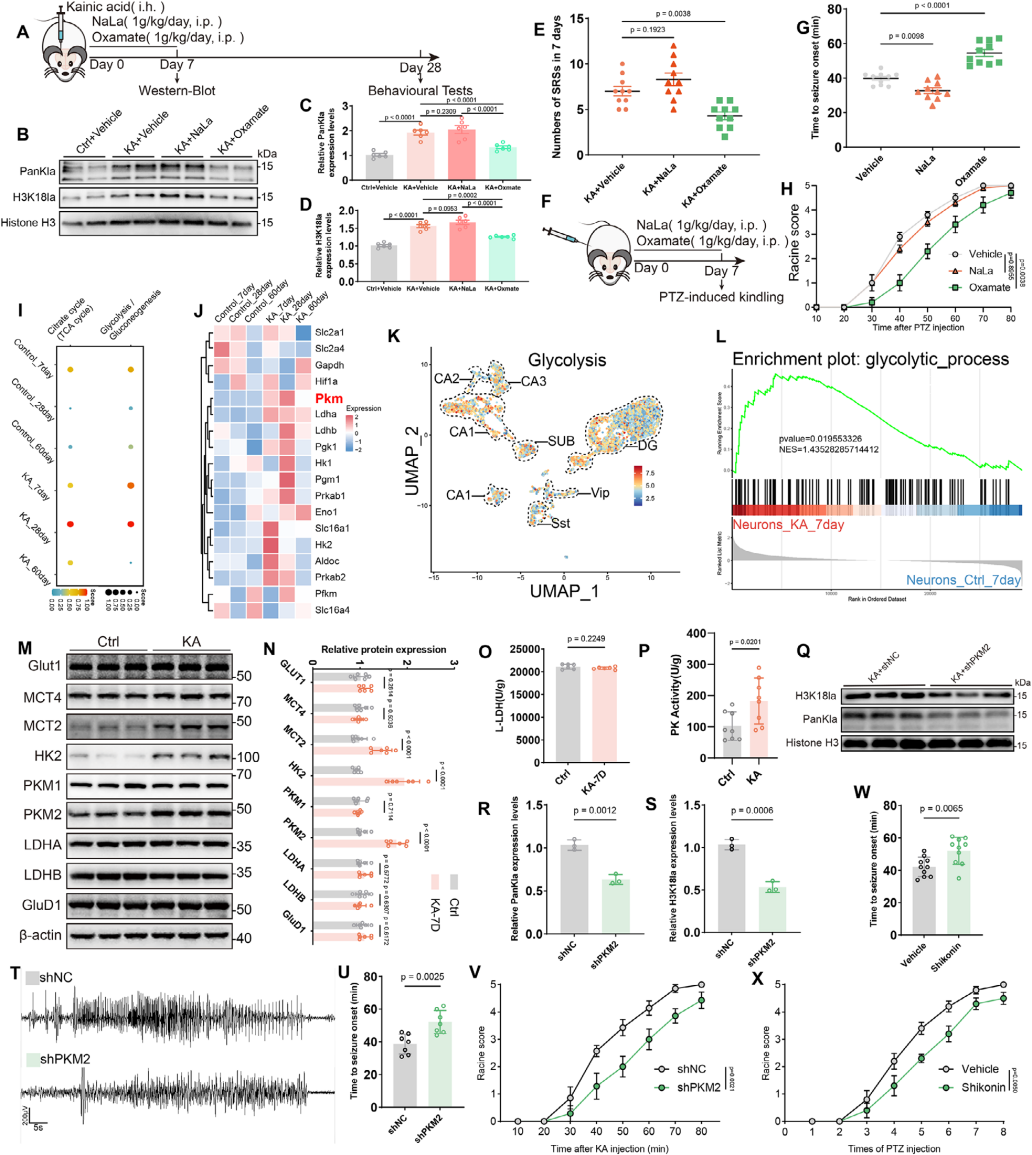
PKM2‐mediated glycolysis drives histone lactylation and enhances seizure susceptibility. (A) Schematic of the metabolic intervention strategy. KA‐injected mice received NaLa (1 g/kg/day, i.p.) or oxamate (1 g/kg/day, i.p.) for 7 days; hippocampal tissue was collected on day 7 for western blot analysis, and behavioral assessments were performed on day 28. (B–D) Representative western blots (B) and quantification of PanKla (C) and H3K18la (D) in hippocampal lysates. Histone H3 was used as a loading control (*n* = 6 mice per group). (E) Numbers of spontaneous recurrent seizure (SRS) events over 7 days of continuous video monitoring in the chronic KA model (*n* = 10 mice per group). (F) Schematic of the acute PTZ kindling paradigm with NaLa or oxamate pre‐treatment. (G and H) Time to seizure onset (G) and Racine scale scores over time (H) following PTZ injection in vehicle‐, NaLa‐, or Oxamate‐pre‐treated mice (*n* = 10 mice per group). (I) Bubble plot from snRNA‐seq showing pathway enrichment scores for TCA cycle and glycolysis/gluconeogenesis gene sets in hippocampal cells from control and KA‐treated mice at 7, 28, and 60 days post‐injection. (J) Heatmap of glycolysis‐related gene expression across time points in hippocampal neurons. (K) UMAP of hippocampal neuronal subtypes colored by glycolytic gene expression score. (L) GSEA enrichment plot for the glycolytic_process gene set in KA‐D7 vs. Ctrl‐D7 neurons. (M,N) Representative western blots (M) and quantification (N) of glycolytic enzymes (GLUT1, MCT4, MCT2, HK2, PKM1, PKM2, LDHA, LDHB, and GLUD1) in hippocampal lysates from control and KA‐D7 mice. β‐Actin was used as a loading control (*n* = 6 mice per group). (O,P) L‐LDH (O) and pyruvate kinase (P) enzymatic activity in hippocampal lysates from control and KA‐treated mice (*n* = 6 and 8 mice per group, respectively). (Q–S) Representative Western blots (Q) and quantification of PanKla (R) and H3K18la (S) in hippocampal lysates from KA+shNC and KA+shPKM2 mice. Histone H3 was used as a loading control (*n* = 3 mice per group). (T,U) Representative EEG traces (T) and latency to seizure onset (U) in shNC and shPKM2 mice following KA injection (*n* = 7 mice per group). Scale bars, 200 µV and 5 s. (V) Racine scale scores over time in shNC and shPKM2 mice following KA injection (*n* = 7 mice per group). (W,X) Time to seizure onset (W) and Racine scale scores across successive PTZ injections (X) in vehicle‐ and shikonin‐treated mice (*n* = 10 mice per group). Data are presented as mean ± SEM. Statistical significance was determined by one‐way ANOVA with Tukey's post hoc test (C–E,G), an unpaired, two‐tailed Student's t‐test (N–P,R,S,U), or two‐way repeated measures ANOVA with Bonferroni's post hoc test (H,V,X). Exact p values are indicated in the figure. See also Figure S3.

Given that H3K18la levels are substantially elevated during epileptogenesis (Figure 1), we next sought to define the upstream metabolic pathways governing this epigenetic mark and its functional contribution to seizure susceptibility. We hypothesized that aberrant lactate metabolism, a known feature of the epileptic brain [36, 37], provides the necessary substrate—lactoyl‐CoA—for histone lactylation [19]. To investigate this, we first pharmacologically manipulated lactate levels in mice (Figure 2A). In KA‐induced epilepsy models, Intraperitoneal administration of sodium lactate (NaLa, 1 g/kg/day) did not increase hippocampal PanKla and H3K18la levels. Conversely, treatment with oxamate(1 g/kg/day), a lactate dehydrogenase (LDH) inhibitor, markedly reduced these epigenetic marks (Figure 2B–D) [17, 22]. In the chronic KA model, exogenous administration of NaLa did not significantly alter the frequency of spontaneous recurrent seizures (SRSs) compared to the vehicle group. In contrast, treatment with oxamate significantly reduced SRS occurrence over the 7‐day monitoring period (Figure 2E). In an acute PTZ‐induced seizure model (Figure 2F), NaLa administration shortened seizure latency, whereas oxamate treatment significantly delayed seizure onset and attenuated progression of seizure severity (Figure 2G,H).

To identify the source of lactate in the epileptic hippocampus, we reanalyzed a single‐nuclei RNA sequencing (snRNA‐seq) dataset of hippocampal tissue from control and KA‐treated mice (GSE298522). Our analysis revealed a distinct transcriptional signature in neurons 7‐and 28‐days post‐KA injection, characterized by a robust activation of glycolysis pathways (Figure 2I,J). Given the role of astrocytes in energy supply in the brain, we also analyzed the glycolysis gene expression profile of astrocytes and found that the expression of glycolysis‐related genes was slightly increased during the critical period of epilepsy (Figure S3A–C). Uniform Manifold Approximation and Projection (UMAP) visualization revealed that this glycolytic signature was localized to distinct neuronal clusters, including CA1, CA2, CA3, subiculum (SUB), and dentate gyrus (DG) populations (Figure 2K). Gene Set Enrichment Analysis (GSEA) of neuronal transcripts at day 7—a critical window for epileptogenesis—showed significant enrichment for the “glycolytic process” gene set (Figure 2L). Among glycolysis‐related genes, pyruvate kinase M (*Pkm*) was among the most prominently upregulated (Figure 2J), consistent with its established role in metabolic reprogramming during pathological states [38, 39, 40, 41].

We validated these transcriptomic findings at the protein and mRNA level. At the mRNA levels, *Pkm2*, and *Hk2* were selectively upregulated in KA‐D7 hippocampus, while *Pkm1* was unchanged (Figure S3D). Western blot analysis of hippocampal lysates from KA‐treated mice confirmed a significant upregulation of key glycolytic enzymes, including the monocarboxylate transporter MCT2, hexokinase 2 (HK2), and pyruvate kinase M2 (PKM2), whereas PKM1 levels remained unaltered (Figure 2M,N). Notably, the increase in PKM2 protein was the most substantial among all assessed glycolytic enzymes, suggesting it may represent a key regulatory node in this metabolic shift during epileptogenesis. Immunofluorescence revealed that HK2 upregulation was confined to Iba1‐positive microglia, whereas PKM2 was predominantly increased in NeuN‐positive neurons (Figure S3E,F), justifying our neuron‐focused mechanistic investigation of PKM2. To distinguish between the contributions of enhanced glycolysis vs. altered lactate conversion, we assessed the enzymatic activities of LDH pyruvate kinase (PK). While total LDH activity remained unchanged in KA‐treated hippocampi (Figure 2O), PK activity was significantly elevated (Figure 2P). Pyruvate levels were also elevated (Figure S3G), consistent with augmented glycolytic flux toward lactate production. These results indicate that lactate accumulation is driven primarily by increased glycolytic flux through PKM2 rather than by altered LDH‐mediated pyruvate‐to‐lactate conversion. Importantly, although total LDH enzymatic activity was not upregulated, the potent anti‐seizure effects of oxamate demonstrate that basal LDH activity remains a critical and necessary step in this pathogenic cascade.

To genetically validate the role of PKM2, we employed adeno‐associated virus (AAV)‐mediated short hairpin RNA (shRNA) to knock down PKM2 specifically in hippocampal neurons in vivo. Knockdown efficiency was confirmed by neuronal fluorescence imaging, western blot, and was accompanied by reductions in PK enzymatic activity and hippocampal lactate levels (Figure S3H–L). PKM2 knockdown significantly reduced both PanKla and H3K18la levels in KA‐treated mice (Figure 2Q–S). Functionally, shPKM2 mice demonstrated significantly prolonged latency to seizure onset following KA injection and markedly attenuated seizure progression compared with shNC controls, as quantified by continuous electroencephalogram (EEG) recording and Racine scale scoring (Figure 2T–V).

Complementary pharmacological inhibition further corroborated these findings. Shikonin, a specific inhibitor of the PKM2 tetramer [42], reduced PK enzymatic activity and lactate levels, and suppressed H3K18la and PanKla levels in vivo (Figure S3M–Q), confirming on‐target PKM2 inhibition. In the PTZ kindling paradigm, shikonin‐treated mice exhibited significantly longer latency to seizure onset and lower Racine scale scores across successive PTZ injections compared with vehicle‐treated controls (Figure 2W,X).

Collectively, these results establish a mechanistic link between metabolic dysregulation and epigenetic modifications central to epileptogenesis. Enhanced glycolytic flux through PKM2 in neurons generates excess lactate, which serves as the substrate for H3K18 lactylation, thereby increasing seizure susceptibility.

### 2.3 H3K18la Promotes Neuronal Dysfunction in KA‐Induced Epileptic Mice via Transcriptional Regulation of COP1

Having established that H3K18la is significantly upregulated during epileptogenesis and functionally linked to altered lactate metabolism, we next sought to identify the downstream transcriptional targets responsible for H3K18la‐mediated seizure susceptibility. To systematically characterize the genome‐wide binding profile of H3K18la, we performed chromatin immunoprecipitation sequencing (ChIP‐seq) using hippocampal tissues from control(sham) and KA‐induced epileptic mice at day 7 post‐injection.

**FIGURE 3:**
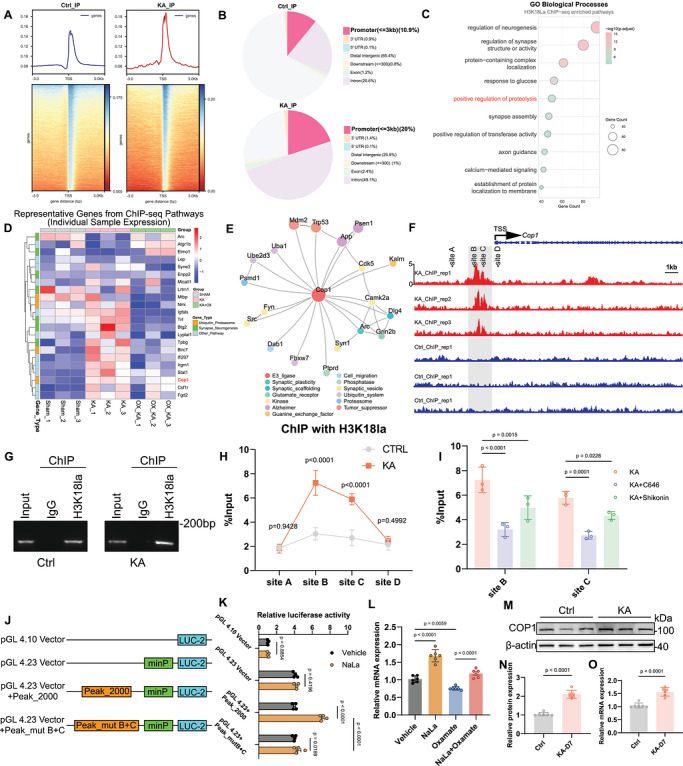
H3K18la targets the *Cop1* promoter in the epileptic hippocampus. (A) ChIP‐seq profile plots showing H3K18la enrichment within ±3 kb of transcription start sites (TSS) in control (Ctrl_IP) and KA‐treated (KA_IP) hippocampal tissue. Heatmaps below display H3K18la signal intensity ranked by peak score. (B) Pie charts showing the genomic distribution of H3K18la ChIP‐seq peaks in Ctrl_IP and KA_IP samples. (C) GO biological process enrichment analysis of genes associated with H3K18la peaks in KA‐treated samples. Bubble size represents gene count; color intensity reflects −log_10_(adjusted p‐value). (D) Heatmap displaying RNA expression levels of representative genes from H3K18la‐enriched pathways across experimental groups (Sham, KA, and KA + Oxamate). Genes are categorized by functional annotation including ubiquitin‐proteasome system, synaptic neurogenesis, and other pathways. Values represent z‐score normalized expression. (E) Protein‐protein interaction network centered on COP1, highlighting its connections to synaptic plasticity genes (blue), ubiquitin system components (yellow), kinases (green), and other functional categories. Node colors represent functional annotations based on STRING database analysis. (F) IGV browser tracks showing H3K18la ChIP‐seq signal at the Cop1 locus in KA and Ctrl replicates. ChIP‐qPCR primer sites (A–D) and the TSS are indicated. Scale bar, 1 kb. (G) Representative agarose gel of ChIP‐PCR amplification products (∼200 bp) from control and KA hippocampal tissue using IgG or anti‐H3K18la antibody, with input DNA as a loading reference. (H) ChIP‐qPCR quantification of H3K18la enrichment at Cop1 promoter sites A–D in control and KA‐D7 mice, expressed as percentage of input (*n* = 3 mice per group). (I) ChIP‐qPCR analysis of H3K18la enrichment at *Cop1* promoter sites B and C in KA‐treated mice co‐administered with vehicle, C646, or shikonin (*n* = 3 mice per group). (J and K) Luciferase reporter assay validating the transcriptional activity of the H3K18la‐enriched *Cop1* promoter region. (J) Schematic of reporter constructs. (K) Relative luciferase activity in vehicle‐ and NaLa‐treated cells transfected with the indicated constructs (*n* = 4 per group). (L) *Cop1* mRNA expression in hippocampal tissue from mice treated with vehicle, NaLa, oxamate, or NaLa + oxamate (*n* = 6 mice per group). (M,N) Representative western blot (M) and quantification (N) of COP1 protein in hippocampal lysates from control and KA‐D7 mice. β‐Actin was used as a loading control (*n* = 6 mice per group). (O) *Cop1* mRNA expression in hippocampal tissue from control and KA‐D7 mice (*n* = 6 mice per group). Data are presented as mean ± SEM. Statistical significance was determined by an unpaired, two‐tailed Student's *t*‐test (H,N,O) or one‐way ANOVA with Tukey's post hoc test (I,K,L). Exact p values are indicated in the figure.

ChIP‐seq analysis revealed distinct chromatin binding patterns between control and epileptic conditions, with H3K18la showing markedly enhanced enrichment around transcription start sites (TSS) in KA‐treated sample compared to control (Figure 3A). Genomic annotation of H3K18la peaks demonstrated a dramatic redistribution of chromatin occupancy during epileptogenesis, with increased binding at promoter regions (20.0% in KA vs. 10.9% in control) (Figure 3B). This redistribution toward gene regulatory elements suggested a key role for H3K18la in transcriptional regulation during epileptogenesis, consistent with its role as an activating histone modification [43, 44, 45].

To understand the functional significance of H3K18la‐regulated genes, we performed gene ontology (GO) analysis of H3K18la‐enriched peaks in epileptic samples. The analysis revealed significant enrichment for biological processes critically involved in neuronal function and epileptogenesis, including “regulation of neurogenesis,” “synapse assembly,” and “positive regulation of proteolysis” (Figure 3C). Additional enriched pathways included “axon guidance,” “calcium‐mediated signaling,” and “protein‐containing complex localization,” collectively pointing to H3K18la's involvement in synaptic plasticity and neuronal connectivity [46, 47]. To pinpoint key effector genes, we integrated our ChIP‐seq data with RNA sequencing (RNA‐seq) performed on hippocampal tissue from sham, KA, and KA+oxamate‐treated mice. This integrated analysis identified a cohort of genes whose transcriptional upregulation correlated with increased H3K18la occupancy at their promoters (Figure 3D). Notably, genes involved in the UPS, including *Cop1, Csf1r*, and *Fgd2*, showed coordinated upregulation that was attenuated by oxamate treatment, confirming the lactate‐dependent nature of this transcriptional program.

Among these candidates, we identified the E3 ubiquitin ligase *Cop1* (Constitutive photomorphogenic protein 1, also known as *Rfwd2*), as a prominent target. COP1 emerged as particularly compelling given its established role in protein degradation pathways and its potential involvement in synaptic protein regulation [31]. Protein‐protein interaction network analysis revealed that *Cop1* is functionally connected to key synaptic plasticity genes, including *Arc, Dlg4, Grin2b*, and *Camk2a*, as well as components of the UPS (Figure 3E). This network topology positions COP1 as a potential hub regulator capable of coordinating protein turnover at synapses during epileptogenesis.

To validate our ChIP‐seq findings, we performed ChIP‐qPCR analysis targeting four distinct regions (sites A–D) within the *Cop1* promoter. ChIP‐seq tracks at the *Cop1* locus clearly showed enhanced H3K18la signal in the KA condition, with peak regions corresponding to our qPCR validation sites (Figure 3F). Agarose gel electrophoresis confirmed the specificity of our ChIP‐qPCR products, showing the expected ∼200 bp amplicons in H3K18la‐immunoprecipitated samples but not in IgG controls (Figure 3G). Consistent with our sequencing data, H3K18la enrichment was significantly increased at site B and site C in KA‐treated mice compared to controls, while sites A and D showed no significant changes (Figure 3H). Although H3K18la enrichment was also detected at the promoters of other UPS‐related genes (*Fbxo4*, *Ube2k*, *Csf1r*, and *Fgd2*), the increase at *Cop1* was the most pronounced (Figure S4A). *Csf1r* mRNA was significantly upregulated (Figure S4B); however, Fgd2 mRNA showed no significant change (Figure S4C), and both genes are predominantly expressed in microglia rather than neurons. COP1 was therefore selected for further investigation based on its established neuronal expression and E3 ubiquitin ligase function. Crucially, the enhanced H3K18la binding at sites B and C was significantly reduced by treatment with C646 (a p300/CBP inhibitor) or shikonin (Figure 3I), confirming that lactate‐driven H3K18la writing is required for this epigenetic modification at the *Cop1* locus.

To test whether H3K18la enrichment at the *Cop1* promoter is sufficient for transcriptional activation, we performed luciferase reporter assays. A construct containing the 2 kb ChIP‐seq peak region (Peak_2000) upstream of a minimal promoter driving luciferase (pGL4.23+Peak_2000) showed significantly elevated luciferase activity compared to the empty vector control (Figure 3J,K). Critically, mutation of the H3K18la‐binding motifs at sites B and C abolished this transcriptional activation; the mutant construct showed no significant response to NaLa treatment (Figure 3K). These results provide functional evidence that H3K18la enrichment at sites B and C of the *Cop1* promoter is both necessary and sufficient for lactate‐driven transcriptional activation.

The enhanced H3K18la binding at the *Cop1* promoter was reflected in increased *Cop1* expression. Consistent with a lactate‐dependent epigenetic mechanism, *Cop1* mRNA levels were significantly elevated by NaLa treatment and suppressed by oxamate or the combination of NaLa+oxamate (Figure 3L). At the protein level, KA‐treated mice showed a corresponding increase in COP1 as determined by western blot (Figure 3M,N), and *Cop1* mRNA was similarly upregulated in KA‐D7 hippocampus (Figure 3O). COP1 protein was elevated in both neurons and microglia but not astrocytes of the KA‐epileptic hippocampus (Figure S4D–I). COP1 was similarly upregulated in cortical tissue from TLE patients, as shown by western blot and NeuN‐colocalized immunofluorescence (Figure S4K–M). In primary hippocampal neurons, COP1 colocalized with both the inhibitory postsynaptic scaffold gephyrin and the excitatory postsynaptic marker PSD95 (Figure S4J), suggesting a broad synaptic presence.

Collectively, these results demonstrate that H3K18la targets the *Cop1* promoter to enhance its transcriptional activity during epileptogenesis, establishing COP1 as a key downstream effector of lactate‐mediated epigenetic regulation in epileptic brain tissue.

### 2.4 Neuronal COP1 Regulates Seizure Susceptibility by Impairing GABAergic Inhibition

Having identified the E3 ubiquitin ligase COP1 as a transcriptional target of H3K18la, we next sought to determine its functional role in seizure pathogenesis. To achieve this, we employed both gain‐and loss‐of‐function approaches in vivo. We generated neuron‐specific overexpression of COP1 by injecting AAV9‐hSyn‐COP1 (AAV9‐COP1) into the hippocampus of wild‐type mice, with an empty AAV9 vector serving as a negative control (AAV9‐NC). Given that both *Cop1* and *Map2* genes are located on mouse chromosome 1, precluding the generation of *Cop1* f/f;*Map2* CreERT2 homozygous mice, we utilized a spatial and temporal approach for loss‐of‐function studies. Specifically, we stereotaxically injected AAV9‐hSyn‐Cre‐EGFP into the hippocampus of 6‐week‐old *Cop1* f/f mice to achieve neuron‐specific conditional knockout of *Cop1* (COP1 cKO), with their corresponding floxed littermates (COP1 f/f) serving as controls [48]. The genetic targeting strategy and successful manipulation of COP1 protein levels in the hippocampus were confirmed and are detailed in Figure S5A–E.

**FIGURE 4:**
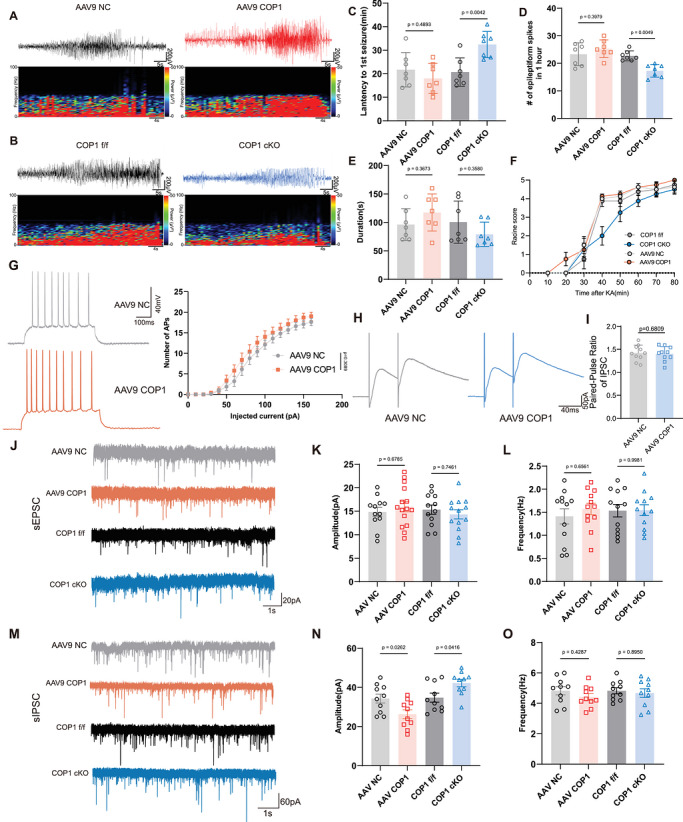
Neuronal COP1 regulates seizure susceptibility through selective modulation of GABAergic inhibition. (A,B) Representative hippocampal EEG traces and power spectrograms during KA‐induced seizures in AAV9‐NC and AAV9‐COP1 mice (A), and COP1 f/f and COP1 cKO mice (B). (C–E) Quantification of latency to first seizure (C), number of epileptiform spikes within 1 h (D), and total seizure duration (E) in AAV9‐NC, AAV9‐COP1, COP1 f/f, and COP1 cKO mice (*n* = 7 mice per group). (F) Seizure severity assessed using the Racine scale over an 80‐min observation period following KA injection in all four groups (*n* = 7 mice per group). (G) Left: representative action potential (AP) traces from CA1 pyramidal neurons in AAV9‐NC and AAV9‐COP1 mice in response to current injection (150 pA). Right: input‐output curves showing AP number across increasing current steps (0–200 pA) (*n* = 7–10 cells from 3 mice per group). (H,I) Representative traces of paired‐pulse evoked IPSC in AAV9‐NC and AAV9‐COP1 mice (H) and quantification of the paired‐pulse ratio at a 50 ms inter‐stimulus interval (I) (*n* = 10–11 cells from 3 mice per group). (J–L) Representative sEPSC traces (J) and quantification of sEPSC amplitude (K) and frequency (L) from CA1 pyramidal neurons in AAV9‐NC, AAV9‐COP1, COP1 f/f, and COP1 cKO mice (*n* = 10–13 cells from 3 mice per group). (M–O) Representative sIPSC traces (M) and quantification of sIPSC amplitude (N) and frequency (O) from the same groups (*n* = 10–13 cells from 3 mice per group). Data are presented as mean ± SEM. Statistical significance was determined by one‐way ANOVA with Tukey's post hoc test (C–E,K,L,N,O), an unpaired, two‐tailed Student's *t*‐test (I), or two‐way repeated measures ANOVA with Bonferroni's post hoc test (F,G). Exact p values are indicated in the figure.

We first assessed seizure susceptibility using the KA model of TLE [49]. Following intraperitoneal KA administration (20 mg/kg), continuous EEG recordings from the hippocampus revealed that COP1 overexpression did not significantly alter seizure latency in AAV9‐COP1 mice compared to AAV9‐NC controls (Figure 4A,C). This lack of effect likely reflects a ceiling in endogenous COP1 activity: because KA‐induced H3K18la strongly upregulates endogenous *Cop1* transcription (Figure 3M–O), additional AAV‐mediated overexpression cannot further increase seizure susceptibility beyond what the endogenous pathway already achieves. Conversely, neuron‐specific deletion of Cop1 conferred significant protection, prolonging the latency to seizure onset in COP1 cKO mice relative to their COP1 f/f counterparts (Figure 4B,C). Consistent with these findings, quantification of epileptiform activity showed that AAV9‐COP1 mice displayed no significant difference in the number of epileptiform spikes within the first hour, while COP1 cKO mice exhibited a significant reduction (Figure 4D). Notably, the total duration of seizure activity was not significantly altered across any of the groups, suggesting that COP1 primarily modulates the threshold for seizure initiation rather than the mechanisms of seizure termination (Figure 4E). Analysis of seizure severity over time using the Racine scale further confirmed that COP1 overexpression did not alter disease progression, whereas its deletion exerted a sustained protective effect (Figure 4F).

To elucidate the cellular mechanisms underlying this phenotype, we performed whole‐cell patch‐clamp recordings in acute hippocampal slices from CA1 pyramidal neurons. Recordings were conducted in magnesium‐free artificial cerebrospinal fluid (ACSF), which removes the voltage‐dependent Mg^2^^+^ block of NMDA receptors and models the hyperexcitable state observed in epilepsy [50]. Overexpression of COP1 did not alter intrinsic neuronal excitability, as the number of action potentials fired in response to depolarizing current injections (0–200 pA) was comparable between AAV9‐COP1 and AAV9‐NC groups (Figure 4G). Furthermore, paired‐pulse facilitation experiments revealed no differences in the paired‐pulse ratio of evoked inhibitory postsynaptic currents (eIPSCs) at a 50 ms inter‐stimulus interval, indicating that COP1 does not regulate presynaptic neurotransmitter release machinery (Figure 4H,I) [51].

Given that epilepsy often arises from an imbalance between synaptic excitation and inhibition [52], we next examined spontaneous postsynaptic currents. Analysis of spontaneous excitatory postsynaptic currents (sEPSCs) revealed no significant differences in either amplitude or frequency between the groups, suggesting that basal glutamatergic transmission was intact (Figure 4J–L). In contrast, we observed a profound and specific deficit in inhibitory signaling. COP1 overexpression in AAV9‐COP1 mice led to a significant decrease in the amplitude of spontaneous inhibitory postsynaptic currents (sIPSCs) compared to controls (Figure 4M,N). Conversely, genetic deletion of Cop1 in cKO neurons resulted in a marked increase in sIPSC amplitude (Figure 4M). The frequency of sIPSCs was unaffected in all conditions, pointing toward a postsynaptic, rather than presynaptic, locus of action (Figure 4O).

Taken together, these data demonstrate that neuronal COP1 is a critical regulator of seizure susceptibility. It enhances network hyperexcitability not by altering intrinsic firing properties or excitatory transmission, but by specifically reducing the strength of postsynaptic GABAergic inhibition. This finding is consistent with previous work establishing COP1 as a regulator of synaptic proteins [30] and highlights a novel mechanism for seizure control.

### 2.5 COP1 Functions as an E3 Ubiquitin Ligase That Targets GABA_A_Rβ2 for Proteasomal Degradation

**FIGURE 5:**
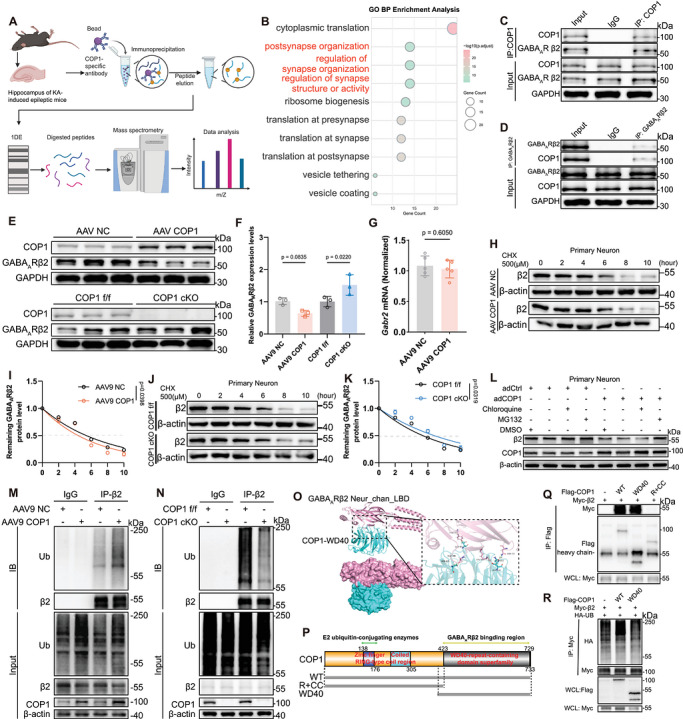
COP1 targets the GABA_A_Rβ2 subunit for proteasomal degradation through its WD40 substrate‐binding domain. (A) Workflow for identifying COP1‐interacting proteins from KA‐induced epileptic hippocampal tissue by co‐immunoprecipitation and mass spectrometry (IP‐MS). Created in BioRender. meng, Y. (2026) https://BioRender.com/impy2u4 (B) GO biological process enrichment analysis of COP1‐interacting proteins identified by IP‐MS. Dot size represents gene count; color intensity reflects −log_10_(adjusted p‐value). (C) Co‐immunoprecipitation (Co‐IP) confirming the physical interaction between COP1 and GABA_A_Rβ2 in KA‐induced epileptic hippocampal lysates. GAPDH in input lanes serves as a loading control. (D) Reciprocal Co‐IP confirming the interaction between endogenous GABA_A_Rβ2 and COP1. (E,F) Representative Western blots (E) and quantification (F) of GABA_A_Rβ2 protein levels in hippocampal lysates from AAV9‐NC, AAV9‐COP1, COP1 f/f, and COP1 cKO mice. GAPDH was used as a loading control (*n* = 3 mice per group). (G) qRT‐PCR analysis of *Gabrb2* mRNA in hippocampal tissue from AAV9‐NC and AAV9‐COP1 mice (*n* = 5 mice per group). (H,I) Cycloheximide (CHX, 500 µm) chase assay in primary neurons transduced with AAV9‐NC or AAV9‐COP1. Representative Western blots (H) and quantification of remaining GABA_A_Rβ2 protein levels over time (I). β‐actin serves as loading control (*n* = 3 independent cultures). (J,K) CHX chase assay in primary neurons from COP1 f/f and COP1 cKO mice. Representative Western blots (J) and quantification of remaining GABA_A_Rβ2 protein levels over time (K). β‐actin serves as loading control (*n* = 3 independent cultures). (L) Representative western blots of GABA_A_Rβ2 and COP1 in primary neurons transduced with adCtrl or adCOP1 and treated with the proteasome inhibitor MG132 (10 µm), chloroquine (CQ, 50 µm), or vehicle (DMSO) for 6 h. β‐Actin serves as a loading control. (M) In vivo ubiquitination assay in hippocampal lysates from AAV9‐NC and AAV9‐COP1 mice. GABA_A_Rβ2 was immunoprecipitated, and ubiquitinated forms were detected by anti‐ubiquitin (Ub) immunoblotting. (N) In vivo ubiquitination assay in hippocampal lysates from COP1 f/f and COP1 cKO mice, as in (M). (O) Structural model of the predicted interaction interface between the COP1 WD40 domain (cyan) and the GABA_A_Rβ2 ligand‐binding domain (magenta), highlighting key interacting residues. (P) Schematic of full‐length COP1 and truncation constructs (WT, R + CC, and WD40) used in domain mapping experiments. (Q) Co‐IP assay in HEK293T cells co‐expressing Myc‐GABA_A_Rβ2 with Flag‐tagged COP1 WT, R + CC, or WD40 constructs. (R) Ubiquitination assay in HEK293T cells co‐expressing Myc‐GABA_A_Rβ2, Flag‐COP1 (WT or WD40), and HA‐ubiquitin. Ubiquitinated GABA_A_Rβ2 was detected by anti‐HA immunoblotting following anti‐Myc immunoprecipitation, Data are presented as mean ± SEM. Statistical significance was determined by one‐way ANOVA with Tukey's post hoc test (F), an unpaired, two‐tailed Student's *t*‐test (G), or two‐way repeated measures ANOVA with Bonferroni's post hoc test (I,K). Exact p values are indicated in the figure.

To elucidate the molecular mechanism underlying COP1's regulation of synaptic function and epileptogenesis, we performed an unbiased immunoprecipitation‐mass spectrometry (IP‐MS) screen using KA‐induced epileptic hippocampal tissue (Figure 5A). GO enrichment analysis demonstrated that COP1‐associated proteins were prominently enriched in biological processes governing synaptic function, including “postsynapse organization,” “regulation of synapse organization,” “regulation of synapse structure or activity,” and “vesicle tethering” (Figure 5B). These findings position COP1 as a central regulator of synaptic protein homeostasis, consistent with its established function as a RING‐finger E3 ubiquitin ligase [53].

From the list of candidate interacting proteins, we focused on the γ‐aminobutyric acid type A receptor β2 subunit (GABA_A_Rβ2), a critical component of the inhibitory synapse. The interaction between COP1 and GABA_A_Rβ2 was confirmed by co‐immunoprecipitation using COP1 as bait, with GAPDH serving as a loading control (Figure 5C), and was further validated by reciprocal co‐immunoprecipitation using GABA_A_Rβ2 as bait (Figure 5D). Consistent with a direct interaction at inhibitory synaptic sites, COP1 and GABA_A_Rβ2 were highly colocalized in primary hippocampal neurons (Pearson's R = 0.83) and in CA1 neurons of KA‐treated mice (R = 0.89), as demonstrated by line‐scan intensity analysis and Pearson's correlation (Figure S5F–K). COP1 also co‐immunoprecipitated with synaptotagmin, synapsin 1, and syntaxin 1b (Figure S6A–C), indicating broader synaptic protein interactions but substrate selectivity for ubiquitination.

To determine the functional consequence of COP1 manipulation on GABA_A_Rβ2 protein levels, we performed western blot analysis across four groups. COP1 overexpression (AAV9‐COP1) led to a significant reduction in GABA_A_Rβ2 protein, while COP1 cKO resulted in a marked accumulation of GABA_A_Rβ2, with GAPDH serving as a loading control (Figure 5E,F). This effect was mediated post‐translationally, as qRT‐PCR analysis showed that *Gabrb2* mRNA levels were unaffected by COP1 overexpression (Figure 5G). Neither COP1 overexpression nor cKO altered the protein levels of syntaxin 1b, synapsin 1, or synaptotagmin (Figure S6D–G), demonstrating that COP1‐mediated ubiquitination specifically targets GABA_A_Rβ2 among its interacting partners.

To investigate whether COP1 affects GABA_A_Rβ2 protein stability, we performed cycloheximide (CHX) chase experiments in primary neurons. Compared to control neurons (AAV9‐NC), COP1 overexpression significantly accelerated the degradation of GABA_A_Rβ2 (Figure 5H,I). Conversely, COP1 cKO significantly extended GABA_A_Rβ2 stability, with protein levels remaining substantially higher than in COP1 f/f controls at all time points examined (Figure 5J,K). Treatment with the proteasome inhibitor MG132, but not the autophagy inhibitor chloroquine (CQ), completely rescued GABA_A_Rβ2 protein levels in COP1‐overexpressing neurons, demonstrating that COP1 promotes GABA_A_Rβ2 degradation specifically via the ubiquitin‐proteasome system (Figure 5L). Consistent with this, COP1 overexpression led to a dramatic increase in polyubiquitinated GABA_A_Rβ2 in epileptic hippocampal tissue (Figure 5M), while COP1 cKO markedly reduced GABA_A_Rβ2 polyubiquitination (Figure 5N), confirming its role in marking the receptor subunit for proteasomal destruction [54].

COP1 is a canonical RING‐finger E3 ligase, whose activity typically relies on a substrate‐recognition domain and a catalytic RING domain that recruits E2‐conjugating enzymes [55]. To dissect the molecular basis of the COP1‐GABA_A_Rβ2 interaction, we generated truncation mutants of COP1: the N‐terminal RING finger and coiled‐coil domains (R + CC) and the C‐terminal WD40 repeat domain (Figure 5P). Computational modeling predicted a stable interaction between the COP1 WD40 domain and the ligand‐binding domain of GABAARβ2, with an optimal docking affinity score of −709.91 (Figure 5O). Co‐immunoprecipitation assays confirmed that the WD40 domain was both necessary and sufficient to bind GABA_A_Rβ2, whereas the R + CC domain was dispensable for this interaction (Figure 5Q). However, the WD40 domain alone, despite its ability to bind the substrate, was unable to induce ubiquitination of GABA_A_Rβ2 (Figure 5R), confirming a classic E3 ligase mechanism in which the WD40 domain serves as a substrate‐binding scaffold and the RING domain confers catalytic activity. Collectively, these data establish that COP1 targets the GABA_A_Rβ2 subunit for UPS‐mediated degradation.

Consistent with these findings, GABA_A_Rβ2 protein levels were significantly reduced in cortical tissue from TLE patients compared with TBI controls (Figure S6H,I), extending the translational relevance of this mechanism to human epilepsy.

### 2.6 COP1 Mediates K48‐Linked Ubiquitination of GABA_A_Rβ2 at Lysine 204 and 216, Reducing Its Synaptic Membrane Abundance

**FIGURE 6:**
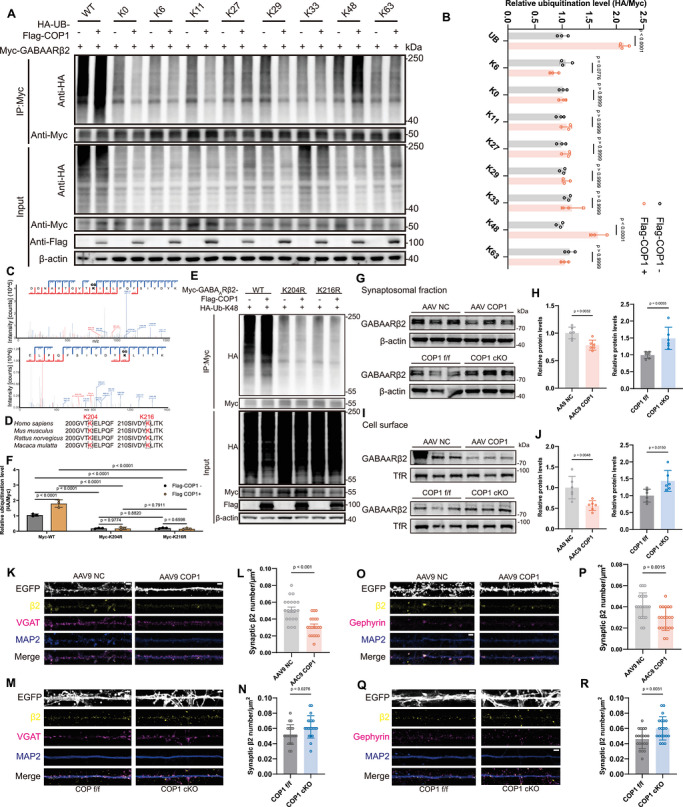
COP1 mediates K48‐linked polyubiquitination of GABA_A_Rβ2 at K204 and K216, reducing its synaptic membrane abundance. (A,B) HEK293T cells were co‐transfected with Myc‐GABA_A_Rβ2, Flag‐COP1, and HA‐tagged ubiquitin constructs bearing single‐lysine mutations (K6, K11, K27, K29, K33, K48, or K63), an all‐lysine‐to‐arginine mutant (K0), or wild‐type ubiquitin (WT). Cell lysates were immunoprecipitated with anti‐Myc antibody and immunoblotted with the indicated antibodies (A). Densitometric quantification of the relative ubiquitination level (HA/Myc) in the presence or absence of Flag‐COP1 is shown in (B) (*n* = 3 independent experiments). (C) MS/MS spectra from mass spectrometry analysis of immunoprecipitated Myc‐GABA_A_Rβ2, identifying K204 (upper) and K216 (lower) as the ubiquitinated lysine residues. (D) Protein sequence alignment of the GABA_A_Rβ2 intracellular loop containing K204 and K216 across *Homo sapiens, Mus musculus, Rattus norvegicus*, and *Macaca mulatta*. (E,F) HEK293T cells were co‐transfected with wild‐type (WT) Myc‐GABA_A_Rβ2 or lysine‐to‐arginine mutants (K204R or K216R), together with Flag‐COP1 and HA‐Ub‐K48. Lysates were immunoprecipitated with anti‐Myc antibody and immunoblotted for the indicated proteins (E). Densitometric quantification of K48‐linked polyubiquitination (HA/Myc) is shown in (F) (*n* = 3 independent experiments). (G,H) Western blot analysis (G) and quantification (H) of GABA_A_Rβ2 protein levels in synaptosomal fractions isolated from epileptic hippocampal tissue of AAV9‐NC or AAV9‐COP1 mice (upper panels), and COP1 f/f or COP1 cKO mice (lower panels). β‐Actin serves as a loading control (*n* = 6 mice per group). (I,J) Western blot analysis (I) and quantification (J) of GABA_A_Rβ2 levels in plasma membrane fractions from the same groups as in (G). Transferrin receptor (TfR) serves as a loading control for membrane fractionation (*n* = 6 mice per group). (K,L) Representative confocal images (K) and quantification of synaptic GABA_A_Rβ2 puncta density (L) from primary hippocampal neurons (DIV14) transduced with AAV9‐NC or AAV9‐COP1, immunostained for EGFP (white), GABA_A_Rβ2 (β2, yellow), VGAT (magenta), and MAP2 (blue). Scale bar, 3 µm (*n* = 3 independent cultures, 10–12 neurons per culture). (M and N) Representative confocal images (M) and quantification of synaptic GABA_A_Rβ2 puncta density colocalizing with VGAT (N) from primary hippocampal neurons derived from COP1 f/f or COP1 cKO mice, immunostained as in (K). Scale bars as in (K) (*n* = 3 independent cultures, 10–12 neurons per culture). (O,P) Representative confocal images (O) and quantification of synaptic GABA_A_Rβ2 puncta density colocalizing with gephyrin (P) from primary hippocampal neurons (DIV14) transduced with AAV9‐NC or AAV9‐COP1, immunostained for EGFP (white), GABA_A_Rβ2 (β2, yellow), gephyrin (magenta), and MAP2 (blue). Scale bars as in (K) (n = 3 independent cultures, 10–12 neurons per culture). (Q,R) Representative confocal images (Q) and quantification (R) from primary hippocampal neurons derived from COP1 f/f or COP1 cKO mice, immunostained as in (O). Scale bars as in (K) (n = 3 independent cultures, 10–12 neurons per culture). Data are presented as mean ± SEM. Statistical significance was determined by one‐way ANOVA with Tukey's post hoc test (B,F) or an unpaired, two‐tailed Student's *t*‐test (H,J,L,N,P,R). Exact p‐values are indicated in the figure.

Given that COP1 drives proteasomal degradation of GABA_A_Rβ2, we sought to elucidate the underlying molecular mechanism. The topology of polyubiquitin chains often dictates the fate of the substrate protein [56]. We therefore aimed to identify the type of ubiquitin linkage catalyzed by COP1 on GABA_A_Rβ2. Co‐immunoprecipitation assays in HEK293T cells, using a panel of linkage‐specific ubiquitin mutants (K6, K11, K27, K29, K33, K48, K63, K0, and WT), revealed that COP1 expression robustly increased K48‐linked polyubiquitination of GABA_A_Rβ2 (Figure 6A). Densitometric quantification confirmed that K48‐linked ubiquitin chains were the predominant modification attributable to COP1, while other linkages including K63 were not significantly elevated (Figure 6B), indicating that COP1 specifically attaches K48‐linked polyubiquitin chains to GABA_A_Rβ2 to signal for its proteasomal degradation [57].

We next used mass spectrometry analysis of immunoprecipitated GABA_A_Rβ2 to map the precise ubiquitination sites. This screen identified K204 and K216 as the ubiquitinated potential lysine residues (Figure 6C). These residues are located within the intracellular loop between transmembrane domains 3 and 4 (TM3‐4), a region known to be critical for receptor trafficking and modulation [58]. Cross‐species sequence alignment revealed that K204 and K216 are highly conserved across mammals, suggesting functional importance (Figure 6D).

To validate these sites, we generated GABA_A_Rβ2 mutants in which these lysines were substituted with arginine (K204R, K216R) to prevent ubiquitination. When co‐expressed with COP1 and K48‐specific ubiquitin (HA‐Ub‐K48), mutation of either K204 or K216 markedly reduced GABA_A_Rβ2 polyubiquitination compared with wild‐type GABAARβ2, as confirmed by immunoprecipitation and densitometric quantification (Figure 6E,F). These results establish K204 and K216 as the primary COP1 target sites on GABA_A_Rβ2.

To investigate the physiological impact of this ubiquitination on GABA_A_Rβ2 subcellular distribution, we isolated synaptosomal and plasma membrane fractions from hippocampal tissue of AAV9‐COP1‐overexpressing mice and neuronal COP1 cKO mice. COP1 overexpression significantly reduced GABA_A_Rβ2 protein levels in synaptosomal fractions, while COP1 cKO conversely increased these levels; densitometric quantification confirmed bidirectional regulation by COP1 (Figure 6G,H). Consistent findings were observed in plasma membrane fractions as assessed by surface biotinylation: COP1 overexpression markedly reduced, and COP1 cKO substantially increased, cell‐surface GABA_A_Rβ2 abundance (Figure 6I,J). Together, these results establish COP1 as a key bidirectional regulator of GABA_A_Rβ2 synaptic and cell‐surface expression.

To assess the functional consequences at inhibitory synapses, we performed immunofluorescence in primary hippocampal neurons. COP1 overexpression dramatically reduced the density of synaptic GABA_A_Rβ2 puncta colocalizing with the vesicular GABA transporter (VGAT), a marker for presynaptic inhibitory boutons (Figure 6K,L). Reciprocally, COP1 cKO neurons exhibited a significant increase in GABAARβ2‐VGAT co‐puncta density compared with COP1 f/f controls (Figure 6M,N), demonstrating that endogenous COP1 tonically suppresses inhibitory synaptic GABA_A_Rβ2 clustering. Parallel analyses using gephyrin, a critical postsynaptic scaffolding protein that anchors GABA_A_Rs at inhibitory synapses, yielded concordant results: COP1 overexpression significantly reduced GABA_A_Rβ2‐gephyrin colocalization (Figure 6O,P), while COP1 cKO neurons showed a corresponding increase in GABA_A_Rβ2‐gephyrin co‐puncta (Figure 6Q,R).

These data collectively demonstrate that COP1‐mediated K48‐linked polyubiquitination at residues K204 and K216 destabilizes GABA_A_Rβ2, reduces its synaptic membrane abundance, and diminishes its clustering at inhibitory postsynaptic densities, thereby reducing inhibitory synaptic strength.

### 2.7 GABA_A_ Receptor β2 Subunit Downregulation Is a Key Mediator of COP1‐Driven Seizure Susceptibility

To confirm that COP1's seizure‐protective effects are specifically mediated through GABA_A_Rβ2 regulation, we tested whether AAV‐mediated knockdown of GABA_A_Rβ2 (shGabrb2) would reverse the protection conferred by neuronal *Cop1* deletion. Four groups were studied: COP1 f/f + scramble vector (COP1 f/f + Vector), COP1 cKO, COP1 f/f + shGABA_A_Rβ2 KD, and COP1 cKO + shGABA_A_Rβ2 KD [59].

**FIGURE 7:**
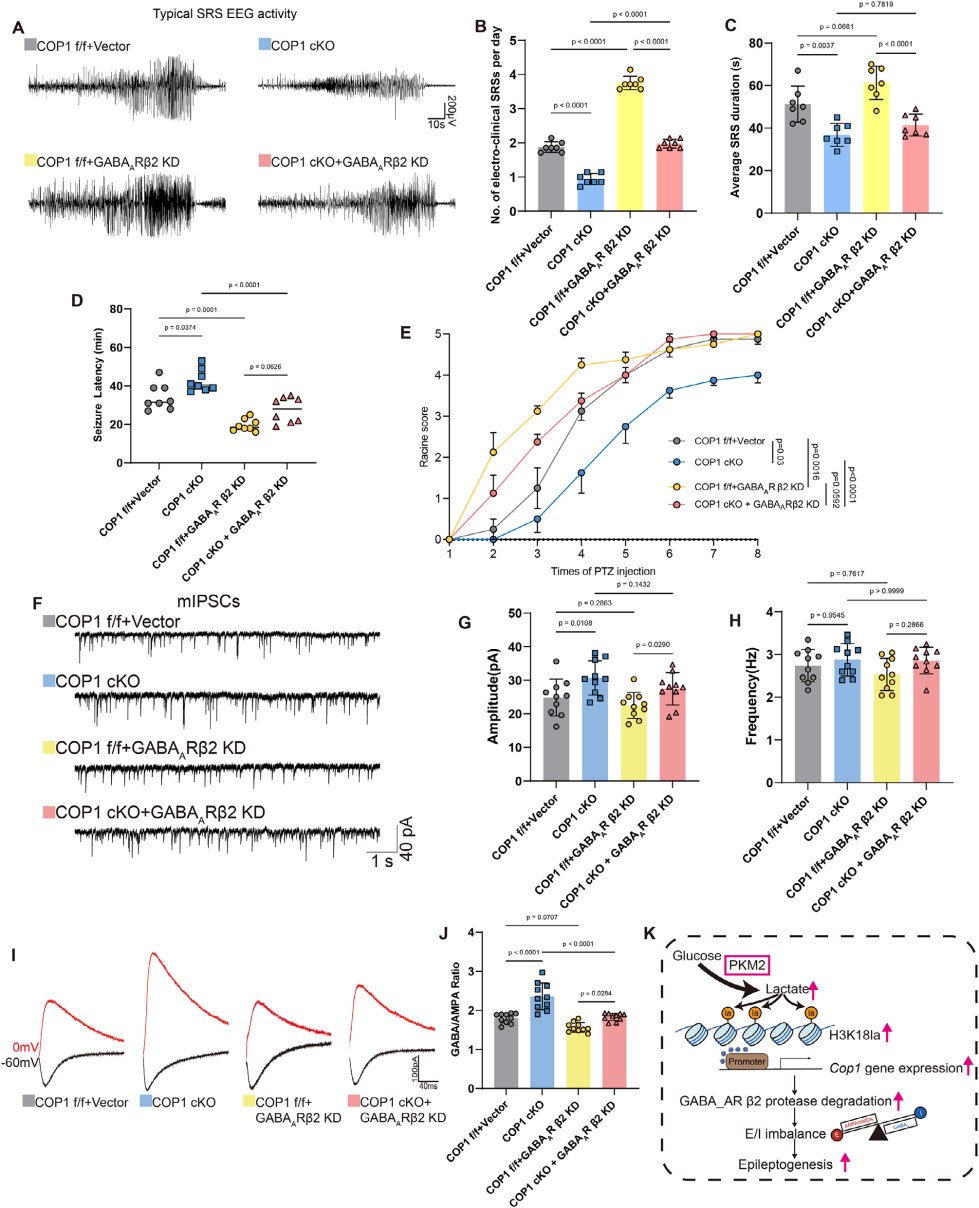
GABAARβ2 knockdown reverses the seizure‐protective effects of neuronal *Cop1* deletion. (A–C) Chronic seizure activity during the SRS phase in KA‐treated mice from four groups: COP1 f/f + Vector, COP1 cKO, COP1 f/f + GABA_A_Rβ2 KD, and COP1 cKO + GABA_A_Rβ2 KD. (A) Representative EEG traces of electro‐clinical SRS events recorded during continuous video‐EEG monitoring. (B) Quantification of daily electro‐clinical SRS frequency (n = 7 mice per group). (C) Average SRS duration (n = 7 mice per group). (D,E) Seizure susceptibility in response to PTZ (50 mg/kg, i.p.) administration in the same four groups. (D) Latency to the first generalized tonic‐clonic seizure (n = 7 mice per group). (E) Seizure severity assessed by the Racine scale across successive PTZ injections (n = 7 mice per group). (F–H) Miniature inhibitory postsynaptic currents (mIPSCs) recorded from CA1 pyramidal neurons in hippocampal slices from all four groups. (F) Representative mIPSC traces. Scale bars, 1 s and 40 pA. Quantification of mIPSC amplitude (G) and frequency (H) (*n* = 12–15 cells from 3 mice per group). (I,J) Assessment of the synaptic excitation/inhibition (E/I) balance. (I) Representative traces of evoked GABAergic (recorded at 0 mV, red) and AMPAergic (recorded at −60 mV, black) currents from all four groups. (J) Quantification of the GABA/AMPA current ratio (*n* = 12–15 cells from 3 mice per group). (K) Schematic diagram summarizing the proposed pathogenic PKM2–lactate–H3K18la–COP1–GABAARβ2 axis in epileptogenesis. Data are presented as mean ± SEM. Statistical significance was determined by one‐way ANOVA with Tukey's post hoc test (B–D,G,H,J) or two‐way repeated measures ANOVA with Bonferroni's post hoc test (E). Exact p values are indicated in the figure.

To evaluate spontaneous seizure burden in a clinically relevant model, we first monitored chronic SRS during the post‐status epilepticus phase by continuous video‐EEG. Compared with COP1 f/f + Vector controls, COP1 cKO mice exhibited markedly fewer electro‐clinical SRS events per day (Figure 7A,B) and shorter average SRS duration (Figure 7C). GABA_A_Rβ2 knockdown in COP1 cKO mice abolished this protection, restoring both SRS frequency and duration to levels comparable to those of COP1 f/f controls (Figure 7B,C). GABA_A_Rβ2 knockdown alone in COP1 f/f mice significantly increased SRS frequency (Figure 7B), but did not significantly alter SRS duration (Figure 7C), indicating that endogenous GABA_A_Rβ2 is important for restraining seizure occurrence.″

We next assessed acute seizure susceptibility by PTZ challenge. COP1 cKO mice showed significantly prolonged latency to the first generalized tonic‐clonic seizure compared with COP1 f/f + Vector controls (Figure 7D). Cumulative seizure severity assessed by the Racine scale across successive PTZ injections was correspondingly reduced in COP1 cKO mice (Figure 7E). GABA_A_Rβ2 knockdown abolished the seizure‐protective effect of neuronal Cop1 deletion, as seizure latency in COP1 cKO + shGABA_A_Rβ2 KD mice was no longer prolonged compared with COP1 f/f + shGABA_A_Rβ2 KD mice (Figure 7D), and Racine scores were restored toward control levels (Figure 7E). This finding aligns with prior evidence that GABA_A_Rβ2 is critical for maintaining inhibitory tone and preventing epileptogenesis [60, 61, 62, 63].

To elucidate the synaptic mechanisms underlying these behavioral differences, we performed whole‐cell patch‐clamp recordings from CA1 pyramidal neurons in hippocampal slices. COP1 cKO mice exhibited a significant increase in miniature inhibitory postsynaptic current (mIPSC) amplitude compared with COP1 f/f + Vector controls, reflecting enhanced postsynaptic GABAergic strength; mIPSC frequency was not significantly altered, consistent with a predominantly postsynaptic locus of effect (Figure 7F–H). GABA_A_Rβ2 knockdown in COP1 cKO mice completely reversed the amplitude increase without affecting frequency (Figure 7G,H), confirming that elevated postsynaptic GABA_A_Rβ2 abundance is the primary driver of enhanced inhibitory transmission in COP1 cKO mice.

Assessment of the synaptic excitation/inhibition (E/I) balance using evoked current recordings confirmed these findings: the GABA/AMPA current ratio was significantly elevated in COP1 cKO neurons relative to COP1 f/f + Vector controls, and this enhancement was fully normalized by GABA_A_Rβ2 knockdown in COP1 cKO mice (Figure 7I,J). These electrophysiological data collectively demonstrate that the seizure‐protective phenotype of COP1 cKO mice is mechanistically dependent on increased GABA_A_Rβ2‐mediated inhibitory synaptic transmission.

The overall proposed mechanism is summarized in Figure 7K. Seizure‐induced PKM2‐driven glycolysis elevates lactate levels, promoting H3K18la at the *Cop1* promoter and increasing COP1 expression. COP1 subsequently drives K48‐linked polyubiquitination and proteasomal degradation of GABA_A_Rβ2, reducing inhibitory synaptic transmission, and creating an E/I imbalance that perpetuates epileptogenesis. Taken together, our findings establish the H3K18la–COP1–GABA_A_Rβ2 axis as a pathogenic metabolic‐epigenetic‐proteostatic pathway in epilepsy and identify each node as a potential therapeutic target for restoring inhibitory synaptic homeostasis.

## 3 Discussion

Our study unveils a previously unrecognized pathogenic pathway in epileptogenesis where enhanced glycolytic flux via PKM2 generates excess lactate, driving H3K18 lactylation that activates transcription of the E3 ubiquitin ligase *Cop1*, ultimately promoting proteasomal degradation of GABA_A_Rβ2 and reducing inhibitory transmission. This metabolic‐epigenetic crosstalk represents a fundamental mechanism linking cellular bioenergetics to synaptic dysfunction in epileptogenesis, and offers new therapeutic targets for the approximately 30% of patients with drug‐resistant epilepsy [6, 8].

The functional prominence of H3K18la among histone lactylation sites observed in our multi‐site profiling is consistent with the established role of H3K18 as a transcriptionally activating residue. Genome‐wide mapping has demonstrated that H3K18la marks active CpG island‐containing promoters and tissue‐specific active enhancers [45]. The abundance of H3K18la was significantly higher than at other sites (H4K12la, H4K16la, and H3K9la) in two independent epilepsy models provides convergent evidence for its status as the functionally dominant lactylation mark in this pathological context. Since Zhang et al. first described histone lactylation as a epigenetic mechanism linking glycolytic state to gene transcription [18], this modification has been implicated in diverse conditions—from immunosuppressive macrophage activity in glioblastoma [64] to microglial activation in Alzheimer's disease and Parkinson's disease [21, 23, 24, 65]. Our work establishes its pivotal role in epilepsy, where lactate accumulation during seizures—reaching concentrations of 5–15 mm in the epileptic focus [13]—exceeds the threshold required for robust histone lactylation. This positions H3K18la as a metabolic sensor that translates acute bioenergetic stress into durable transcriptional changes, providing a molecular substrate for the clinical observation that seizures beget seizures [66]. Notably, exogenous NaLa administration did not further elevate H3K18la in KA‐treated mice despite the clear anti‐lactylation effect of oxamate, most likely reflecting saturation of the endogenous lactylation machinery in the epileptic state, where intraneuronal lactate levels are already sufficient to maximally drive H3K18la. Alternatively, peripheral NaLa may not efficiently access the relevant nuclear histone compartment due to blood‐brain barrier transport constraints or intracellular metabolic processing; this interpretation remains tentative and warrants direct investigation.

Our findings position neurons as a primary site of pathological lactate generation during epileptogenesis—an observation that warrants consideration in light of the classical Astrocyte‐Neuron Lactate Shuttle (ANLS) model. The ANLS proposes that astrocytes are the predominant glycolytic cell type under physiological conditions, supplying lactate to neurons as an energetic substrate [34]. However, accumulating evidence demonstrates that neurons can undergo substantial aerobic glycolysis under pathological conditions—including ischemia, traumatic brain injury, and seizures—independently of astrocytic supply [67, 68]. Our snRNA‐seq data, showing selective upregulation of glycolytic genes including Pkm2 in neuronal clusters with concurrent PKM2 protein induction specifically in NeuN‐positive neurons, are consistent with this pathological neuronal glycolytic shift. It should be noted, however, that these transcriptomic observations are correlational and do not directly quantify metabolic flux. Cell‐type‐specific isotope tracing experiments will be required to definitively map the contribution of neuronal vs. astrocytic lactate to histone lactylation in vivo.

Our rationale for focusing mechanistic investigations on PKM2, rather than the comparably upregulated HK2, is supported by cell‐type‐specific localization data. HK2 upregulation in the KA model was predominantly confined to Iba1‐positive microglia, whereas PKM2 induction was concentrated in NeuN‐positive neurons. Given that the epileptogenic mechanism we characterize—H3K18la‐driven COP1 transcription, GABA_A_Rβ2 ubiquitination, and impairment of inhibitory synaptic transmission—is fundamentally neuronal in nature, PKM2 represents the more proximate and cell‐type‐relevant upstream driver of the pathway. HK2 induction in microglia may nonetheless contribute to neuroinflammatory metabolic reprogramming and represents an independent avenue for future investigation. Our enzymatic data also merit clarification regarding the respective roles of PKM2 and LDH in lactate production. Although total LDH enzymatic activity was not significantly elevated in the epileptic hippocampus, this finding does not imply that LDH is dispensable for the observed lactate surge. Rather, basal LDH activity is sufficient—and indeed necessary—to convert the markedly increased pyruvate generated by elevated PKM2‐driven PK activity into lactate, making the two enzymes complementary rather than redundant therapeutic targets within the same metabolic pathway. This model is entirely consistent with the robust seizure protection conferred by the LDH inhibitor oxamate [14] and the anti‐seizure drug stiripentol, which was recently identified as an LDH inhibitor [17].

The identification of *Cop1* as the critical transcriptional target of H3K18 lactylation reveals an unexpected convergence of metabolic signaling and protein quality control at inhibitory synapses. While COP1 has been extensively characterized as a tumor suppressor and negative regulator of p53 stability [69], it also functions as an autism susceptibility gene that induces synaptic deficits in the prefrontal cortex [31] and promotes dendritic spine development in cortical neurons [30]. Our demonstration that COP1 directly promotes K48‐linked polyubiquitination and proteasomal degradation of GABA_A_Rβ2 provides a mechanism for activity‐dependent remodeling of inhibitory transmission. This finding gains particular significance in light of the fact that mutations in GABA_A_R β‐subunits represent one of the most common genetic causes of epilepsy, with over 150 pathogenic variants identified to date [61]. Importantly, the clinical relevance of this pathway is substantiated by our analysis of human tissue: we observed significantly elevated H3K18la, increased COP1 expression, and reduced GABA_A_Rβ2 protein levels in cortical specimens from drug‐resistant TLE patients compared with TBI controls, establishing that the PKM2–lactate–H3K18la–COP1–GABA_A_Rβ2 cascade is operative in the human epileptic brain.

The observation that H3K18la is elevated in microglia—but not astrocytes—in the epileptic hippocampus, corroborated in human TLE tissue, merits dedicated consideration. Microglia undergo profound metabolic reprogramming during neuroinflammation, shifting toward aerobic glycolysis in a manner analogous to the Warburg effect, which would be expected to increase intracellular lactate availability and consequently drive H3K18la. In macrophages, histone lactylation has been shown to regulate pro‐inflammatory gene transcription programs [18, 64], and in the context of brain aging, H3K18la in senescent microglia has been linked to NF‐κB pathway activation and neuroinflammatory amplification. By extension, elevated microglial H3K18la in epilepsy may similarly enhance the expression of neuroinflammatory mediators that amplify seizure susceptibility [23]. Microglial inflammatory activation is a well‐established contributor to epileptogenesis [5], and the cell‐type‐specific elevation of H3K18la in neurons and microglia—with sparing of astrocytes—suggests fundamentally distinct lactate utilization and nuclear delivery pathways across brain cell types. Future studies employing microglial‐specific genetic tools and single‐cell metabolic profiling will be essential to delineate the precise functional consequences of microglial H3K18la in epilepsy.

Our findings illuminate how metabolic adaptation becomes pathologically entrenched through epigenetic mechanisms. The PKM2–lactate–H3K18la axis represents a form of metabolic memory whereby transient glycolytic bursts during seizures leave lasting marks on chromatin architecture. This concept aligns with emerging evidence that metabolic intermediates function as cofactors for chromatin‐modifying enzymes, creating a link between cellular metabolism, and epigenetic state [70]. In epilepsy, this metabolic‐epigenetic coupling may explain the progressive nature of the disorder and the failure of symptomatic treatments to alter disease trajectory. Indeed, the efficacy of metabolic interventions such as the ketogenic diet may derive partly from disrupting this pathological cascade—β‐hydroxybutyrate not only serves as an alternative fuel but also functions as an endogenous histone deacetylase (HDAC) inhibitor that may compete with lactate for histone modification sites, thereby attenuating the pathological H3K18la response [71, 72].

Clinical translation of these findings will require careful consideration of therapeutic windows and biomarker development. The temporal dynamics of H3K18la accumulation suggest that intervention during the latent period following brain injury could prevent epileptogenesis, though identifying at‐risk patients remains challenging. Liquid biopsy approaches detecting circulating lactate or cell‐free chromatin bearing H3K18la marks could provide non‐invasive monitoring of pathway activation. PKM2 represents a particularly attractive therapeutic target given the existence of well‐characterized inhibitors developed for oncological applications, though optimization for central nervous system (CNS) penetration remains necessary [73]. Histone lactyltransferases, principally p300/CBP in their lactylation‐competent configuration, represent an orthogonal epigenetic target—the emerging development of isoform‐selective p300 inhibitors offers a potential strategy [19]. Furthermore, COP1 E3 ligase activity itself constitutes a downstream effector node amenable to pharmacological intervention; small molecules disrupting the COP1‐GABA_A_Rβ2 substrate interaction at the WD40 domain interface could provide synapse‐level precision in restoring inhibitory transmission. Monitoring GABA_A_Rβ2 surface abundance as a surrogate biomarker of pathway activation may additionally facilitate patient stratification and therapeutic response assessment.

### 3.1 Limitations of the Study

Several important limitations warrant consideration in interpreting our findings. First, while our promoter luciferase assays and genetic perturbation experiments provide functional evidence that H3K18la enrichment at sites B and C of the Cop1 promoter promotes transcriptional activation, establishing strict single‐mark causality through site‐specific histone mutation remains technically challenging with current methodologies [74, 75]. The p300/CBP inhibitor C646 reduces both H3K18la and H3K18ac, and therefore the individual contribution of H3K18la—as distinguished from co‐occurring H3K18 acetylation—cannot yet be formally assigned without site‐specific epigenome editing tools. Future investigations employing dCas9‐based targeted epigenetic modifiers to manipulate a single modification at a defined locus will be required to provide more definitive causal validation [76]. Second, our analyses focused primarily on H3K18la without fully examining potential crosstalk with other modifications at this residue. H3K18 serves as a substrate for multiple post‐translational modifications including acetylation (H3K18ac) and methylation (H3K18me), which may compete or cooperate with lactylation in regulating chromatin structure [77]. The dynamic interplay between these marks and their collective impact on transcriptional outcomes warrants further investigation. Third, although lactoyl‐CoA has been recently confirmed as the proximal cofactor for histone lactylation [20], direct mass spectrometric quantification of lactoyl‐CoA in brain tissue and stable‐isotope (^1^ ^3^C‐lactate) tracing to demonstrate site‐specific incorporation at histone H3K18 were technically unfeasible with currently available platforms. The causal assignment of increased lactate as the direct precursor for H3K18la therefore relies on convergent pharmacological and genetic perturbation evidence rather than direct metabolic tracing, which remains an important validation goal for future mechanistic studies. Fourth, while our snRNA‐seq and immunofluorescence data implicate neurons as the primary site of pathway activation, contributions from astrocytic lactate shuttling or microglial metabolic reprogramming cannot be excluded, as the transcriptomic data are correlational and do not directly quantify metabolic flux at cellular resolution. Cell‐type‐specific isotope labeling approaches will be required to map lactate flux with the necessary precision. Fifth, our in vivo experiments were conducted exclusively in male C57BL/6J mice. This choice was deliberate: estrogen and progesterone exert opposing and complex effects on seizure threshold—estrogen being predominantly proconvulsant and progesterone anticonvulsant through allosteric potentiation of GABA_A_Rs via its neuroactive metabolite allopregnanolone—and their cyclical fluctuations introduce substantial within‐subject variability in epilepsy models that would confound the mechanistic interpretation of our findings [78, 79]. Investigating how sex and gonadal hormones interact with the PKM2–H3K18la–COP1–GABA_A_Rβ2 axis represents an important and clinically relevant direction for future research, particularly given the phenomenon of catamenial epilepsy. Finally, while we have validated the key components of our proposed pathway in human cortical and hippocampal specimens from drug‐resistant TLE patients undergoing surgical resection, these findings are restricted to a single epilepsy etiology. Confirmation in additional clinical contexts—including genetic epilepsies, post‐traumatic epilepsy, and pediatric epilepsy syndromes—will be essential to establish the generalizability of these translational observations.

## 4 Conclusion

The PKM2–lactate–H3K18 lactylation–COP1–GABA_A_Rβ2 pathway represents a fundamental advance in our understanding of epileptogenesis, revealing how transient metabolic reprogramming during seizures becomes durably encoded through epigenetic mechanisms. Operating at three interconnected levels—metabolic, epigenetic‐transcriptional, and proteostatic—this axis establishes lactate as a critical signaling molecule that bridges cellular bioenergetics and transcriptional control, with pathological consequences that extend far downstream to inhibitory synaptic dysfunction. The conservation of elevated H3K18la, COP1 upregulation, and GABA_A_Rβ2 loss in human drug‐resistant TLE tissue underscores the translational relevance of our findings. By identifying multiple pharmacologically actionable nodes—PKM2, histone lactyltransferases (p300/CBP), COP1, and GABA_A_Rβ2 surface stability—our study offers a mechanistic foundation for developing next‐generation epilepsy therapeutics that target root metabolic causes of seizure susceptibility, rather than merely managing symptomatic manifestations.

## 5 Methods

### 5.1 Animals

All animal experiments were conducted in strict accordance with the guidelines of the Ethics and Use Committee of Chongqing Medical University (Approval No. IACUC‐CQMU‐2024‐10051). *Cop1* floxed mice (*Cop1*‐Flox; Catalog No: NM‐CKO‐2103287), on a C57BL/6J background, were generated by Shanghai Model Organisms Center, Inc. (Shanghai, China). In this model, loxP sites flank exons 3–5 of the *Cop1* gene.

Initial attempts to generate homozygous COP1 f/f; MAP2‐CreERT2 mice for neuron‐specific knockout were unsuccessful due to the syntenic location of the *Cop1* and *Map2* genes on mouse chromosome 1, which impeded viable recombination. Therefore, an alternative viral approach was employed. To achieve neuron‐specific conditional knockout, 6‐week‐old COP1 f/f mice received stereotactic injections of AAV9‐hSyn‐Cre into the hippocampus. Corresponding COP1 f/f mice injected with a control AAV served as controls. Subsequent experiments were conducted 3 weeks post‐injection.

For primary cell cultures, neonatal (P0) C57BL/6J pups of both sexes were used. All animals were housed under a 12‐h light/dark cycle at a controlled temperature of 22 C ± 2°C and humidity of 50% ± 10%, with ad libitum access to food and water.

### 5.2 Viral Vectors

The following adeno‐associated viruses (AAVs) were used in this study: AAV9‐hSyn‐EGFP‐CRE, AAV9‐hSyn‐EGFP‐COP1, AAV9‐hSyn‐EGFP‐NC (negative control), AAV‐hSyn‐shGABRB2‐mCherry (shRNA targeting Gabrb2), and AAV‐hSyn‐shCtrl‐ mCherry (scramble shRNA control). The shRNA GABRB2 sequence was 5′‐GCAGCTGAGAAAGCTGCTAAT‐3′. The scramble shRNA sequence was 5′‐CGCTGAGTACTTCGAAATGTC‐3′, which has no target in the mouse. All viral vectors (titer: ∼1 × 10^1^ ^2^ vg/mL) were produced and provided by BrainVTA (BrainVTA Co., Ltd., Wuhan, China).

### 5.3 Stereotactic Surgery and Virus Injection

Mice were anesthetized with isoflurane (3% induction, 1.5% maintenance) and secured in a stereotaxic frame (RWD Life Science, Shenzhen, China). For viral‐mediated gene delivery, AAVs were injected bilaterally into the hippocampal CA1 region at the following coordinates: Anteroposterior (AP) −2.0 mm, Mediolateral (ML) ±1.5 mm, and Dorsoventral (DV) −1.8 mm, relative to bregma. A volume of 1000 nL per site was infused at a rate of 100 nL/min using a microliter injection pump (Nanoliter 2010, WPI). The injection needle was left in place for an additional 10 min post‐infusion to prevent backflow and ensure diffusion. Animals were allowed to recover for 3–4 weeks before subsequent experiments.

### 5.4 Primary Hippocampal Neuron Culture

Cortical and hippocampal tissues from C57BL/6J mice pups at postnatal day 0 were carefully isolated from neonatal mice, transferred to ice‐cold D‐Hank's balanced salt solution (Solarbio; Beijing, China), dissected into small fragments, and digested with 0.125% trypsin (Gibco) for 10 min at 37°C. The digestion was stopped by adding modified DMEM containing 10% FBS and 1% P/S. After further washing, the tissue was gently dissociated using Pasteur pipettes of decreasing diameter. The cell suspension was pelleted and plated on 6‐well cell culture plates coated with poly‐D‐lysine (Sigma Aldrich). After 4 h, the DMEM was replaced with neurobasal medium containing 2% B27 supplement, 1% P/S, and 1% L‐glutamine (Gibco). Neurons were cultured at 37°C with 5% CO2. Half of the fresh neurobasal medium was replaced every 3 days. Neurons were used for experiments between 12 and 14 DIV.

### 5.5 Epilepsy Models and Pharmacological Interventions

Two distinct methods were used to induce epileptic seizures.

Kainic Acid (KA)‐Induced Seizure Models

#### 5.5.1 Acute Seizure Induction

To induce acute status epilepticus, mice received a single intraperitoneal (i.p.) injection of kainic acid (KA; Abcam, ab120100) at a dose of 20 mg/kg.

#### 5.5.2 Chronic Spontaneous Recurrent Seizures (SRS)

To establish a model of temporal lobe epilepsy with SRS, mice received a stereotactic intra‐hippocampal injection of KA (1 mm in 50 nL saline) into the right hippocampus. Seizure behavior was monitored continuously for 4 h post‐injection and scored according to the Racine scale: (1) mouth and facial movements; (2) head nodding; (3) forelimb clonus; (4) rearing with forelimb clonus; (5) rearing and falling with generalized tonic‐clonic seizures. Only mice exhibiting stage 4–5 seizures were included in subsequent experiments.

#### 5.5.3 Pentylenetetrazol (PTZ)‐Induced Acute Seizure Model

To assess seizure susceptibility, mice were administered an i.p. injection of PTZ (Sigma–Aldrich, P6500) at a convulsive dose of 50 mg/kg. The latency to the first generalized seizure (Racine stage IV and V) was recorded.

#### 5.5.4 Pharmacological Interventions

For metabolic modulation studies, mice received daily i.p. injections of sodium lactate (NaLa; 1 g/kg; Sigma–Aldrich, 71718), the lactate dehydrogenase inhibitor oxamate (1 g/kg; Sigma–Aldrich, O2751), or the PKM2 inhibitor shikonin (5 mg/kg; MCE, HY‐N0691) for 7 consecutive days, commencing on the day of KA administration. Control animals received an equivalent volume of saline.

### 5.6 Electroencephalogram (EEG) Recording and Analysis

Under isoflurane anesthesia, mice were implanted with a stainless‐steel screw electrode over the right hippocampus (AP ‐2.0 mm, ML +1.5 mm from bregma) and a reference electrode over the cerebellum. The assembly was secured to the skull with dental cement. After a 7‐day recovery period, mice were connected to a PINNACLE 3‐CHANNEL TETHERED SYSTEMS (Pinnacle Technology, Inc.) for continuous video‐EEG recordings. EEG signals were acquired and analyzed offline using PINNACLE SIRENIA SEIZURE PRO software. Epileptiform spikes were defined as sharp waveforms with an amplitude at least twice the baseline and duration greater than 5s.

### 5.7 Electrophysiological Recordings

#### 5.7.1 Slice Preparation

Mice (8–10 weeks old) were anesthetized with Pentobarbital Sodium (50 mg/kg) and Perfused Transcardially with an Ice‐Cold Sucrose Solution Containing (in mm) 206 Sucrose, 11 D‐Glucose, 2.5 KCl, 1 NaH_2_PO_4_, 10 MgCl_2_, 2 CaCl_2_ and 26 NaHCO_3_, saturated with 95% O_2_/5% CO_2_. Brains were rapidly removed and transverse hippocampal sections (300 µm) were prepared using a Leica VT 1200S Vibratome. Slices were allowed to recover for 1 h at 32°C in an oxygenated artificial cerebrospinal fluid (ACSF) containing (in mm): 122 NaCl, 3 KCl, 10 D‐Glucose, 1.25 NaH_2_PO_4_, 2 CaCl_2_, 1.3 MgCl_2_, and 26 NaHCO_3_. Slices were then maintained at room temperature until recording.

#### 5.7.2 Whole‐Cell Recordings

Recordings were performed in a submerged chamber maintained at 32°C with constant ACSF perfusion (444 mL/min). Whole‐cell recordings were made from CA1 pyramidal neurons, identified visually using an upright microscope (Zeiss Axioskop 2 FS Plus) with IR‐DIC optics. Borosilicate glass pipettes (3–5 MΩ) were used. Data were recorded with a MultiClamp 700B amplifier, filtered at 10 kHz, and digitized at 20 kHz with a Digidata 1440A interface (Axon Instruments).

For Current‐Clamp Recordings, the Internal Solution Contained (in mm) 60 K_2_SO_4_, 60 NMG, 40 HEPES, 4 MgCl_2_, 0.5 BAPTA, 12 phosphocreatine, 2 Na_2_ATP, and 0.2 Na_3_GTP (pH 7.3, 275–290 mOsm). D‐APV (50 µm), DNQX (20 µm), and picrotoxin (100 µm) were added to block synaptic transmission. A current step protocol was used to evoke action potentials by injecting 500 ms long depolarizing current steps of increasing amplitude from −100 to 300 pA (△20 pA).

For spontaneous excitatory postsynaptic currents (sEPSCs), the internal solution contained (in mm): 130 CsMeSO_3_, 6 CsCl, 1 MgCl_2_, 10 HEPES, 0.3 EGTA, 10 Tris‐Phosphocreatine, 4 Mg‐ATP, and 0.3 Na‐GTP. sEPSCs were recorded in ACSF containing 100 µm picrotoxin.

For spontaneous inhibitory postsynaptic currents (sIPSCs), a high‐Cl^−^ internal solution was used, containing (in mm): 70 CsMeSO_3_, 35 CsCl, 15 TEA‐Cl, 1 MgCl_2_, 0.2 CaCl_2_, 10 HEPES, 0.3 EGTA, 10 Tris‐Phosphocreatine, 4 Mg‐ATP, and 0.3 Na‐GTP. sIPSCs were recorded in ACSF containing 10 µm NBQX and 50 µm APV.

For miniature currents (mIPSCs, mEPSCs), 1 µm tetrodotoxin (TTX) was added to the ACSF.

The amplitudes and frequencies of sIPSCs and sEPSCs were detected by continuous recording for 300 s by using MiniAnalysis software (Synaptosoft).

### 5.8 Mass Spectrometry Analysis

Total protein was extracted from hippocampal tissue samples 7 days after KA injection. COP1‐beads were used for immunoprecipitation of proteins interacting with COP1. The collected interacting proteins were loaded onto SDS‐PAGE. After electrophoresis, the gel was stained with Coomassie blue for 30 min at room temperature. For mass spectrometry analysis, lanes without protein bands on the gel were excised. Mass spectrometric analysis was performed by Applied Protein Technology (Shanghai, China). To identify the ubiquitination modification site of GABA_A_Rβ2, HEK293T cells were transfected with Flag‐COP1, HA‐Ub‐K48, and Myc‐GABA_A_Rβ2 plasmids. After 48 h of transfection, cells were lysed with lysis buffer, and the next steps were as described previously. KEGG pathway enrichment analysis was performed using the DAVID bioinformatics web server (https://david.ncifcrf.gov/) by uploading the gene lists from our mass spectrometric analysis.

### 5.9 qPCR

Total RNA was extracted from epileptic hippocampal tissue samples using RNA simple Total RNA Kit (TIANGEN) according to the manufacturer's instructions. qPCR analysis was performed using the ChamQ Universal SYBR qPCR Master Mix (Vazyme). Gene expression levels were normalized to the housekeeping gene β‐actin. The sequences of qPCR primers are listed in Table S1.

### 5.10 Synaptosome Preparation

All steps were performed at 4°C or on ice. Dissected hippocampi were homogenized in Syn‐PER Reagent (Thermo Scientific; ∼1 mL per 100 mg tissue) containing protease and phosphatase inhibitors. Homogenates were centrifuged at 1200 × g for 10 min. The resulting supernatant was collected and centrifuged at 15 000 × g for 20 min. The pellet (synaptosomes) was resuspended in a buffer containing 3 mm sucrose and 6 mm Tris (pH 8.0) with 1% SDS, briefly sonicated, and stored at −80°C. Protein concentration was quantified by the BCA Protein Assay Kit (Beyotime, P0012).

### 5.11 Plasma Membrane Fraction

Dissected hippocampi were rinsed with ice‐cold PBS, and isolation of the plasma membrane and its associated proteins was conducted with the Minute Plasma Membrane Protein Isolation Kit (Invent Biotech) according to the manufacturer's instructions. The purification of the fractions was analyzed by SDS‐PAGE followed by western blotting with the indicated antibodies.

### 5.12 Biochemical Assays

#### 5.12.1 Lactate Level Measurement

Hippocampal tissues were homogenized in ice‐cold PBS. Intracellular lactate levels in the supernatant were measured using a Lactic Acid Assay Kit (Solarbio, BC2230) according to the manufacturer's protocol. Absorbance was measured at 570 nm, and concentration was calculated from a standard curve.

#### 5.12.2 Enzyme Activity Assays

The enzymatic activities of pyruvate kinase (PK) and lactate dehydrogenase (LDH) in hippocampal homogenates were determined using a Pyruvate Kinase Activity Assay Kit (Abcam, ab83432) and a Lactate Dehydrogenase Activity Assay Kit (Sigma‐Aldrich, MAK066), respectively, following the manufacturers' instructions.

### 5.13 Western Blotting

Cells or hippocampal tissues were lysed in RIPA buffer (Beyotime, P0013B) supplemented with protease and phosphatase inhibitor cocktails (MCE). Protein concentration was determined using a BCA Protein Assay Kit (Beyotime, P0012). Equal amounts of protein (20–40 µg) were resolved by SDS‐PAGE and transferred to polyvinylidene difluoride (PVDF) membranes (Millipore). Membranes were blocked for 1 h with 5% non‐fat milk in TBST and incubated overnight at 4°C with primary antibodies. The primary antibodies used were: anti‐H3K18la (PTM Biolabs, PTM‐1406RM, 1:1000), anti‐H3K14la (PTM Biolabs, 1:1000), anti‐H3K9la (PTM Biolabs, 1:1000), anti‐H4K12la (PTM Biolabs, 1:1000), anti‐H4K16la (PTM Biolabs, 1:1000), anti‐H4K5la (PTM Biolabs, 1:1000), anti‐Pan Kla (PTM Biolabs, 1:1000), anti‐Histone H3 (Proteintech, 1:2000), anti‐PKM2 (Proteintech, 1:1000), anti‐LDHA (Proteintech, 1:1000), anti‐LDHB (Proteintech, 1:1000), anti‐HK2 (Abcam, 1:1000), anti‐GLUT1 (Proteintech, 1:1000), anti‐MCT4 (Proteintech, 1:1000), anti‐MCT2 (Proteintech, 1:1000), anti‐COP1 (Abcam, ab104996, 1:1000), anti‐GABA_A_Rβ2 (NOVUS, 1:500), anti‐Syntaxin 1b (Synaptic Systems, 1:5000), anti‐Synaptotagmin (Synaptic Systems, 1:5000), anti‐Synapsin 1 (Proteintech, 1:1000), anti‐ubiquitin (Proteintech, 1:1000), anti‐MYC tag (Proteintech, 1:1000), anti‐DYKDDDDK tag (Proteintech, 1:1000), anti‐HA tag (Proteintech, 1:1000), anti‐TfR (Abcam, 1:1000), anti‐β‐actin (Proteintech, 66009‐1‐Ig, 1:5000), and anti‐GAPDH (Proteintech, 60004‐1‐Ig, 1:5000). After incubation with HRP‐conjugated secondary antibodies (Jackson ImmunoResearch, 1:5000), protein bands were visualized using ECL detection reagent from BIOSHARP. Images were captured with the FUSION FX SPECTRA/DBT system (VILBER), and band intensities were quantified using Image Lab software.

### 5.14 Dot Blot and Peptide Competition Assay

For the dot blot, synthetic peptides corresponding to H3K18la‐modified, H3K18ac‐modified, or unmodified (PBS) histone H3 lysine 18 sequences were spotted in graded amounts (10, 20, 50, 100, and 500 ng per dot) onto a nitrocellulose membrane. After air‐drying for 30 min at room temperature, the membrane was blocked with 5% non‐fat milk in TBST for 1 h and then incubated overnight at 4°C with either the anti‐H3K18la antibody (1:1000) or the anti‐H3K18ac antibody (1:1000), followed by HRP‐conjugated secondary antibody and ECL detection. For the peptide competition assay, the anti‐H3K18la antibody was pre‐incubated for 1 h at room temperature with PBS (vehicle control), a 20‐fold molar excess of H3K18ac peptide, or a 10‐ or 20‐fold molar excess of H3K18la peptide before incubation with hippocampal lysates from control and KA‐D7 mice. Immunoblotting was performed as described above using total histone H3 as a loading control.

### 5.15 Immunofluorescence

Mice were transcardially perfused with saline followed by 4% paraformaldehyde (PFA). Brains were post‐fixed and cryoprotected in 30% sucrose. Coronal sections (30 µm) were cut using a cryostat (Leica CM1950). Sections or fixed primary neurons were permeabilized with 0.3% Triton X‐100, blocked with 5% normal goat serum, and incubated overnight at 4°C with primary antibodies: anti‐H3K18la (1:100), anti‐NeuN (Millipore, 1:500), anti‐Iba1 (Oasis biofarm, 1:500), anti‐GFAP (CST, #12389, 1:400), anti‐COP1 (Abcam, 1:100), anti‐PSD95 (Abcam, 1:100), anti‐GABA_A_Rβ2 (NOVUS, 1:100), anti‐VGAT (Synaptic Systems, 1:100), anti‐Gephyrin (Synaptic Systems, 147 011, 1:100). Samples were then incubated with Alexa Fluor 488‐, 649‐, or 594‐conjugated secondary antibodies (Invitrogen, 1:500). Coverslips were mounted with DAPI‐containing medium (Vector Laboratories, H‐1200). Images were acquired with a Leica SP8 confocal microscope and analyzed using ImageJ/Fiji software.

### 5.16 Co‐Immunoprecipitation and Ubiquitination Assays

For ubiquitination assays, HEK293T cells were co‐transfected with plasmids encoding Myc‐GABA_A_Rβ2, Flag‐COP1, and HA‐Ubiquitin. Cells were treated with 10 µM MG132 (Selleckchem, S2619) for 6 h before harvesting. Cells were lysed in buffer containing 1% SDS and boiled to denature proteins. After dilution, immunoprecipitation was performed using an anti‐Myc antibody, followed by immunoblotting with anti‐HA or antibodies. For endogenous protein interactions, hippocampal tissue lysates were immunoprecipitated with an anti‐GABA_A_Rβ2 or anti‐COP1 antibody, followed by immunoblotting.

### 5.17 Chromatin Immunoprecipitation (ChIP)

#### 5.17.1 ChIP‐Sequencing

Hippocampal tissues were cross‐linked with 1% formaldehyde for 10 min at room temperature and quenched with glycine (125 mM, 5 min). Chromatin was sonicated to yield fragments of 100–500 bp. Chromatin from three independent biological replicates per group (total: 6 libraries; *n* = 3 control, *n* = 3 KA‐D7) was immunoprecipitated overnight at 4°C with anti‐H3K18la antibody (PTM Biolabs, PTM‐1406RM, 5 µg per sample) or control IgG. Following immunoprecipitation, reverse cross‐linking, and DNA purification, sequencing libraries were prepared using the NEBNext Ultra II DNA Library Prep Kit (NEB, E7645) according to the manufacturer's instructions. Libraries were sequenced on an Illumina NovaSeq 6000 platform with 150 bp paired‐end reads.

Sequencing quality control and alignment. Raw reads were assessed with FastQC (v0.11.9) and adapter sequences were trimmed using Trim Galore (v0.6.7). High‐quality reads were aligned to the mouse reference genome (GRCm38/mm10) using Bowtie2 (v2.4.4) with default parameters. Only uniquely mapped reads were retained. PCR duplicates were removed using Picard MarkDuplicates (v2.26.0). Each library achieved a minimum sequencing depth of ≥20 million uniquely mapped reads. Library complexity was assessed by non‐redundant fraction (NRF ≥ 0.8) and PCR bottleneck coefficient (PBC1 ≥ 0.9), consistent with ENCODE quality standards. The fraction of reads in peaks (FRiP score) was calculated for each library and all samples met a minimum FRiP threshold of 0.01. Pearson correlation coefficients between biological replicates were computed from 500 bp genomic bins using deepTools (v3.5.1) bamCoverage and plotCorrelation; all replicate pairs showed R ≥ 0.90.

Peak calling and differential enrichment. Peaks were called using MACS2 (v2.2.7.1) against matched input chromatin controls with a q‐value cutoff of < 0.05. Peaks overlapping blacklisted genomic regions (ENCODE mm10 blacklist) were excluded. Reproducible peaks were identified using the IDR (Irreproducibility Discovery Rate) framework with an IDR threshold of 0.05. Differential H3K18la enrichment between KA and control groups was quantified using DiffBind (v3.2.7) with FDR < 0.05 and fold change ≥ 1.5 as thresholds for significance. Signal tracks (bigWig format) were generated by deepTools bamCoverage with RPKM normalization for visualization in the Integrative Genomics Viewer (IGV, v2.16).

Data availability. All raw sequencing data (FASTQ files) and processed data (peak files, bigWig tracks) have been deposited at the NCBI Gene Expression Omnibus (GEO) under accession number GSE326007 and will be made publicly available upon manuscript acceptance.

#### 5.17.2 ChIP‐qPCR

Hippocampi were removed quickly, snap frozen, and stored at −80°C until further processing. Chromatin immunoprecipitation (ChIP) was performed using the Simple ChIP Plus Enzymatic Chromatin IP Kit (Magnetic Beads (Cell Signaling Technology, #9005) as described by the manufacturer with minor modifications. In brief, samples were finely minced and cross‐linked with 1.5% formaldehyde for 10 min at room temperature and quenched by the addition of glycine for 5 min. Samples were then homogenized to create a single cell suspension. Chromatin was sheared via incubation with 0.5 µL Micrococcal Nuclease at 37°C for 20 min and then sonicated (a cycle of 100W, 1 s on/5 s off; 10 cycles) to break the nuclear membrane. For immunoprecipitation, the diluted chromatin was incubated with 5 µL normal IgG (control), 5 µL H3K18la (PTM Biolabs, PTM‐1406RM), antibodies overnight at 4°C by constant rotation, followed by incubation with 30 µL of Protein G Magnetic Beads for an additional 2 h. Then, DNA was eluted from the beads and purified, and samples were subjected to RT‐qPCR as described above using the indicated primer shown in Appendix Table S1. ChIP‐qPCR results were calculated as the percentage of input DNA.

### 5.18 Luciferase Reporter Assay

A genomic fragment spanning the H3K18la‐enriched region of the Cop1 promoter (sites B and C; approximately −1500–−500 bp relative to the TSS) was cloned upstream of the firefly luciferase coding sequence in the pGL4.23‐Basic vector (Promega). A mutant construct in which the putative H3K18la‐responsive element was deleted served as a negative control. HEK293T cells were seeded in 24‐well plates and co‐transfected with the firefly luciferase reporter plasmid (500 ng per well) and a Renilla luciferase normalization vector (pRL‐TK, 50 ng per well) using Lipofectamine 3000 (Thermo Fisher Scientific) according to the manufacturer's instructions. At 24 h post‐transfection, cells were treated with sodium lactate (NaLa, 20 mM) or an equivalent volume of vehicle (PBS) for an additional 24 h. Cells were then lysed with Passive Lysis Buffer and luciferase activities were measured using the Dual‐Luciferase Reporter Assay System (Promega) on a luminometer. All firefly luciferase values were normalized to Renilla luciferase activity. Data are expressed as relative luciferase units (RLU), normalized to the vehicle‐treated empty vector control.

### 5.19 RNA‐Sequencing Analysis

Total RNA isolated from hippocampus was used for RNA‐seq analysis. cDNA library construction and sequencing were performed by Illumina Novaseq 6000 (LC‐Bio, China) platform. High‐quality reads were aligned to the mouse reference genome using Bowtie2. Expression abundance and variations for each of the genes were normalized to fragments per kilobase of transcript per million mapped reads (FPKM) using RNA‐seq by Expectation Maximization (RSEM). We identified differentially expressed genes (DEGs) between samples and performed clustering analysis and functional annotation. For genes, a fold change of ≥ 1.2 and a false discovery rate (FDR) of < 0.05 were considered statistically significant. Pathways overrepresented by DEGs were annotated in the KEGG (Kyoto Encyclopedia of Genes and Genomes) database.

### 5.20 Single Nucleus RNA Sequencing (snRNA‐seq) Analysis

The snRNA‐seq data of epileptic mice hippocampal samples and normal control (NC) samples, accession numbers GSE298522, were downloaded from the Genomics Expression Omnibus (GEO) database. Data processing was performed using the Cell Ranger pipeline (v6.0). Downstream analysis, including normalization, clustering, and differential gene expression, was conducted using the Seurat R package (v4.0).

To delineate neuronal subpopulations, neurons were extracted from the global cell cluster and subjected to independent re‐clustering. Excitatory neuronal subtypes were identified as follows: subiculum (SUB) neurons by Cdh20, Cobl, and Tshz3; proximal subiculum (ProS) neurons by Arhgap6 and Mpped1; CA1 neurons by Ndst3, Hcn1, Etl4, and Pex5l; CA2 neurons by Asph, Map3k15, and Rgs14; CA3 neurons by Ccbe1, Nectin3, Trhde, and Trps1; and dentate gyrus (DG) neurons by Prox1, Stxbp6, Maml2, and Sema5a. Inhibitory interneuron subtypes were identified by the following canonical markers: somatostatin‐expressing (Sst) interneurons by Sst; vasoactive intestinal peptide‐expressing (Vip) interneurons by Vip; Lamp5‐expressing interneurons by Lamp5; Sncg‐expressing interneurons by Ntng1; and Cajal‐Retzius (CR) cells by Dach1 and Ndnf. Glycolytic gene expression scores were calculated for each neuronal subcluster using the AddModuleScore function in Seurat, based on a curated gene set comprising established glycolysis pathway genes (GO:0006096). Differential glycolytic activity across conditions and subclusters was visualized using UMAP embedding and heatmap representations.

### 5.21 Quantification and Statistical Analysis

All data are presented as mean ± standard error of the mean (SEM). Statistical analyses were performed using GraphPad Prism 9.5. For comparisons between two groups, a two‐tailed unpaired Student's *t*‐test was used. For comparisons involving more than two groups, one‐way or two‐way analysis of variance (ANOVA) followed by Tukey's or Bonferroni's post hoc test was employed. A p‐value of < 0.05 was considered statistically significant. Specific statistical tests and sample sizes (n) for each experiment are detailed in the corresponding figure legends.

## Author Contributions

F.X. supervised the whole project and provided funding. F.X. reviewed and edited the manuscript. Y.M., Y.G., L.J., J.L. designed and performed most of the experiments, analyzed the data, and wrote the manuscript. P.K. performed experiments in mice and analysis of EEG records. Y.L. performed experiments in cells Z.Y. performed bioinformation analysis of ChIP‐seq and snRNA‐seq. R.D. performed the LC‐MS analysis. J.L. performed the genetic analysis. All authors discussed and commented on the manuscript.

## Ethics statement

The use of human brain tissue was approved by the Ethics Committee of the First Affiliated Hospital of Chongqing Medical University (Approval No. 2025‐934‐01). All procedures involving human brain tissue were conducted in accordance with the Declaration of Helsinki. Written informed consent was obtained from all patients (or their legal guardians) prior to tissue collection. Tissue specimens were obtained from patients with drug‐resistant temporal lobe epilepsy (TLE) who underwent surgical resection, and from traumatic brain injury (TBI) patients who served as controls. Detailed clinical and demographic information for all human subjects is provided in Table S2. Patient identifiers were anonymized before tissue processing.

## Conflicts of Interest

The authors declare no conflicts of interest.

## Supporting information




**Supporting File 1**: advs75813‐sup‐0001‐SuppMat.docx.

**Supporting File 2**: advs75813‐sup‐0002‐TableS1‐S3.zip.

**Supporting File 3**: advs75813‐sup‐0003‐Figures.zip.

## References

[1] R. D. Thijs , R. Surges , T. J. O'Brien , and J. W. Sander , “Epilepsy in Adults,” The Lancet 393 (2019): 689–701, 10.1016/S0140-6736(18)32596-0. 30686584

[2] O. Devinsky , A. Vezzani , T. J. O'Brien , et al., “Epilepsy,” Nature Reviews Disease Primers 4 (2018): 18024, 10.1038/nrdp.2018.24. 29722352

[3] R. Köhling and J. Wolfart , “Cold Spring Harb,” Perspectives in Medicine 6 (2016): a022871. 27141079 10.1101/cshperspect.a022871 PMC4852798

[4] C. Bonansco and M. Fuenzalida , “Plasticity of Hippocampal Excitatory‐Inhibitory Balance: Missing the Synaptic Control in the Epileptic Brain,” Neural Plasticity 2016 (2016): 1–13, 10.1155/2016/8607038. PMC4783563 27006834

[5] A. Vezzani , J. French , T. Bartfai , and T. Z. Baram , “The Role of Inflammation in Epilepsy,” Nature Reviews Neurology 7 (2011): 31–40, 10.1038/nrneurol.2010.178. 21135885 PMC3378051

[6] P. Kwan , A. Arzimanoglou , A. T. Berg , et al., “Definition of Drug Resistant Epilepsy: Consensus Proposal by the Ad Hoc Task Force of the ILAE Commission on Therapeutic Strategies,” Epilepsia 51 (2010): 1069–1077, 10.1111/j.1528-1167.2009.02397.x. 19889013

[7] Z. Chen , M. J. Brodie , D. Liew , and P. Kwan , “Treatment Outcomes in Patients With Newly Diagnosed Epilepsy Treated With Established and New Antiepileptic Drugs,” JAMA Neurology 75 (2018): 279, 10.1001/jamaneurol.2017.3949. 29279892 PMC5885858

[8] J. M. Rho and D. Boison , “The Metabolic Basis of Epilepsy,” Nature Reviews Neurology 18 (2022): 333–347, 10.1038/s41582-022-00651-8. 35361967 PMC10259193

[9] Z. Gábor and W. S. Kunz , “Mitochondrial Dysfunction and Seizures: The Neuronal Energy Crisis,” The Lancet Neurology 14 (2015): 956–966, 10.1016/S1474-4422(15)00148-9. 26293567

[10] S. Rowley , M. Patel , and F. Radic , “Mitochondrial INVOLVEMENT and Oxidative Stress in Temporal Lobe Epilepsy,” Biologie Medicale 62 (2013): 121. 10.1016/j.freeradbiomed.2013.02.002 PMC4043127 23411150

[11] M. J. During , I. Fried , P. Leone , A. Katz , and D. D. Spencer , “Direct Measurement of Extracellular Lactate in the Human Hippocampus During Spontaneous Seizures,” Journal of Neurochemistry 62 (1994): 2356–2361, 10.1046/j.1471-4159.1994.62062356.x. 8189240

[12] C. Idil , W. S. Kasoff , M. P. Cassaday , et al., “Extracellular Metabolites in the Cortex and Hippocampus of Epileptic Patients,” Annals of Neurology 57 (2005): 226–235, 10.1002/ana.20380. 15668975

[13] J. Detour , C. Bund , C. Behr , et al., “Metabolomic Characterization of Human Hippocampus From Drug‐Resistant Epilepsy With Mesial Temporal Seizure,” Epilepsia 59 (2018): 607–616, 10.1111/epi.14000. 29341105

[14] D. Skwarzynska , H. Sun , I. Kasprzak , S. Sharma , J. Williamson , and J. Kapur , “Glycolytic Lactate Production Supports Status Epilepticus in Experimental Animals,” Annals of Clinical and Translational Neurology 10 (2023): 1873–1884, 10.1002/acn3.51881. 37632130 PMC10578888

[15] L. Bozzo , J. Puyal , and J.‐Y. Chatton , “Lactate Modulates the Activity of Primary Cortical Neurons Through a Receptor‐Mediated Pathway,” PLoS ONE 8 (2013): 71721, 10.1371/journal.pone.0071721. PMC3741165 23951229

[16] D. Skwarzynska , H. Sun , J. Williamson , I. Kasprzak , and J. Kapur , “Glycolysis Regulates Neuronal Excitability Via Lactate Receptor, HCA1R,” Brain 146 (2023): 1888–1902, 10.1093/brain/awac419. 36346130 PMC10411940

[17] N. Sada , S. Lee , T. Katsu , T. Otsuki , and T. Inoue , “Targeting LDH Enzymes With a Stiripentol Analog to Treat Epilepsy,” Science 347 (2015): 1362–1367, 10.1126/science.aaa1299. 25792327

[18] D. Zhang , Z. Tang , H. Huang , et al., “Metabolic Regulation of Gene Expression by Histone Lactylation,” Nature 574 (2019): 575–580, 10.1038/s41586-019-1678-1. 31645732 PMC6818755

[19] R. Zhu , X. Ye , X. Lu , et al., “ACSS2 acts as a Lactyl‐CoA Synthetase and Couples KAT2A to Function as a Lactyltransferase for Histone Lactylation and Tumor Immune Evasion,” Cell Metabolism 37 (2025): 361–376.e7, 10.1016/j.cmet.2024.10.015. 39561764

[20] R. Liu , X. Ren , Y. E. Park , et al., “Nuclear GTPSCS Functions as a Lactyl‐CoA Synthetase to Promote Histone Lactylation and Gliomagenesis,” Cell Metabolism 37 (2025): 377–394.e9, 10.1016/j.cmet.2024.11.005. 39642882 PMC11798710

[21] R.‐Y. Pan , L. He , J. Zhang , et al., “Positive Feedback Regulation of Microglial Glucose Metabolism by Histone H4 Lysine 12 Lactylation in Alzheimer's Disease,” Cell Metabolism 34 (2022): 634–648.e6, 10.1016/j.cmet.2022.02.013. 35303422

[22] H. Hagihara , H. Shoji , H. Otabi , et al., “Protein Lactylation Induced by Neural Excitation,” Cell Reports 37 (2021): 109820, 10.1016/j.celrep.2021.109820. 34644564

[23] L. Wei , X. Yang , J. Wang , et al., “H3K18 Lactylation of Senescent Microglia Potentiates Brain Aging and Alzheimer's Disease Through the NFκB Signaling Pathway,” Journal of Neuroinflammation 20 (2023): 208, 10.1186/s12974-023-02879-7. 37697347 PMC10494370

[24] Q. Qin , D. Wang , Y. Qu , et al., “Enhanced Glycolysis‐Derived Lactate Promotes Microglial Activation in Parkinson's Disease via Histone Lactylation,” npj Parkinson's Disease 11 (2025): 3. 10.1038/s41531-024-00858-0 PMC11698869 39753581

[25] B. Bingol and E. M. Schuman , “Activity‐Dependent Dynamics and Sequestration of Proteasomes in Dendritic Spines,” Nature 441 (2006): 1144–1148, 10.1038/nature04769. 16810255

[26] J. Gu , P. Ke , H. Guo , et al., “KCTD13‐Mediated Ubiquitination and Degradation of GluN1 Regulates Excitatory Synaptic Transmission and Seizure Susceptibility,” Cell Death & Differentiation 30 (2023): 1726–1741, 10.1038/s41418-023-01174-5. 37142655 PMC10307852

[27] Y. Wang , H. Yang , N. Li , et al., “A Novel Ubiquitin Ligase Adaptor PTPRN Suppresses Seizure Susceptibility through Endocytosis of Na_V_1.2 Sodium Channels,” Advanced Science 11 (2024): 2400560. 38874331 10.1002/advs.202400560 PMC11304301

[28] A. Ndoja , R. Reja , S.‐H. Lee , et al., “Ubiquitin Ligase COP1 Suppresses Neuroinflammation by Degrading c/EBPβ in Microglia,” Cell 182 (2020): 1156–1169.e12, 10.1016/j.cell.2020.07.011. 32795415

[29] J. T. Glessner , K. Wang , G. Cai , et al., “Autism Genome‐Wide Copy Number Variation Reveals Ubiquitin and Neuronal Genes,” Nature 459 (2009): 569–573, 10.1038/nature07953. 19404257 PMC2925224

[30] G. Zhong , Z. Fang , T. Sun , et al., “Ubiquitin Ligase RFWD2 Promotes Dendritic Spine and Synapse Formation by Activating the ERK/PEA3/c‐Jun Pathway in Rat Cerebral Cortical Neurons,” Biochimica et Biophysica Acta (BBA)—Molecular Basis of Disease 1870 (2024): 167319, 10.1016/j.bbadis.2024.167319. 38909848

[31] Y.‐X. Li , Z.‐N. Tan , X.‐H. Li , et al., “Increased Gene Dosage of RFWD2 Causes Autistic‐Like Behaviors and Aberrant Synaptic Formation and Function in Mice,” Molecular Psychiatry 29 (2024): 2496–2509, 10.1038/s41380-024-02515-7. 38503925 PMC11412905

[32] A. Ksendzovsky , M. Bachani , M. Altshuler , et al., “Chronic Neuronal Activation Leads to Elevated Lactate Dehydrogenase A Through the AMP‐Activated Protein Kinase/Hypoxia‐Inducible Factor‐1α Hypoxia Pathway,” Brain Communications 5 (2023): fcac298, 10.1093/braincomms/fcac298. 36655171 PMC9838803

[33] L. Hu , Y. Liu , Z. Yuan , et al., “Glucose‐6‐Phosphate Dehydrogenase Alleviates Epileptic Seizures by Repressing Reactive Oxygen Species Production to Promote Signal Transducer and Activator of Transcription 1‐mediated N‐methyl‐d‐Aspartic Acid Receptors Inhibition,” Redox Biology 74 (2024): 103236, 10.1016/j.redox.2024.103236. 38875958 PMC11225908

[34] P. J. Magistretti and I. Allaman , “Lactate in the Brain: From Metabolic End‐Product to Signalling Molecule,” Nature Reviews Neuroscience 19 (2018): 235–249, 10.1038/nrn.2018.19. 29515192

[35] R. D. Nass , B. Zur , C. E. Elger , S. Holdenrieder , and R. Surges , “Acute Metabolic Effects of Tonic‐Clonic Seizures,” Epilepsia Open 4 (2019): 599–608, 10.1002/epi4.12364. 31819916 PMC6885665

[36] X. Kuang , S. Chen , and Q. Ye , “The Lactate Metabolism and Protein Lactylation in Epilepsy,” Frontiers in Cellular Neuroscience 18 (2024): 1464169, 10.3389/fncel.2024.1464169. 39876842 PMC11772370

[37] X. Chen and X. Zhu , “Lactate: Beyond a Mere Fuel in the Epileptic Brain,” Neuropharmacology 266 (2025): 110273, 10.1016/j.neuropharm.2024.110273. 39719259

[38] H. R. Christofk , M. G. Vander Heiden , M. H. Harris , et al., “The M2 Splice Isoform of Pyruvate Kinase is Important for Cancer Metabolism and Tumour Growth,” Nature 452 (2008): 230–233, 10.1038/nature06734. 18337823

[39] Y. Zhao , Y. Wang , Y. Wu , et al., “PKM2‐Mediated Neuronal Hyperglycolysis Enhances the Risk of Parkinson's Disease in Diabetic Rats,” Journal of Pharmaceutical Analysis 13 (2023): 187–200, 10.1016/j.jpha.2022.11.006. 36908857 PMC9999299

[40] B. Lian , J. Zhang , X. Yin , et al., “SIRT1 Improves Lactate Homeostasis in the Brain to Alleviate Parkinsonism Via Deacetylation and Inhibition of PKM2,” Cell Reports Medicine 5 (2024): 101684, 10.1016/j.xcrm.2024.101684. 39128469 PMC11384727

[41] L. Lu , H. Wang , X. Liu , et al., “Pyruvate Kinase Isoform M2 Impairs Cognition in Systemic Lupus Erythematosus by Promoting Microglial Synaptic Pruning via the β‐Catenin Signaling Pathway,” Journal of Neuroinflammation 18 (2021): 229, 10.1186/s12974-021-02279-9. 34645459 PMC8513209

[42] H. Zhu , H. Zhang , X.‐J. Zhao , et al., “Tetramerization of PKM2 Alleviates Traumatic Brain Injury by Ameliorating Mitochondrial Damage in Microglia,” Journal of Neuroimmune Pharmacology 19 (2024): 48, 10.1007/s11481-024-10138-6. 39196455

[43] X. Lin , Y. Lei , M. Pan , et al., “Augmentation of Scleral Glycolysis Promotes Myopia Through Histone Lactylation,” Cell Metabolism 36 (2024): 511–525.e7, 10.1016/j.cmet.2023.12.023. 38232735

[44] J. Yu , P. Chai , M. Xie , et al., “Histone Lactylation Drives Oncogenesis by Facilitating m6A Reader Protein YTHDF2 Expression in Ocular Melanoma,” Genome Biology 22 (2021): 85, 10.1186/s13059-021-02308-z. 33726814 PMC7962360

[45] E. Galle , C.‐W. Wong , A. Ghosh , et al., “H3K18 Lactylation Marks Tissue‐Specific Active Enhancers,” Genome Biology 23 (2022): 207, 10.1186/s13059-022-02775-y. 36192798 PMC9531456

[46] А. V. Zaitsev , D. V. Amakhin , A. V. Dyomina , et al., “Synaptic Dysfunction in Epilepsy,” Journal of Evolutionary Biochemistry and Physiology 57 (2021): 542–563, 10.1134/S002209302103008X.

[47] D. J. Englot , L. B. Hinkley , N. S. Kort , et al., “Global and Regional Functional Connectivity Maps of Neural Oscillations in Focal Epilepsy,” Brain 138 (2015): 2249–2262, 10.1093/brain/awv130. 25981965 PMC4840946

[48] B. E. Deverman , P. L. Pravdo , B. P. Simpson , et al., “Cre‐Dependent Selection Yields AAV Variants for Widespread Gene Transfer to the Adult Brain,” Nature Biotechnology 34 (2016): 204–209, 10.1038/nbt.3440. PMC5088052 26829320

[49] M. Lévesque and M. Avoli , “The Kainic Acid Model of Temporal Lobe Epilepsy,” Neuroscience & Biobehavioral Reviews 2013, 37, 2887–2899, 10.1016/j.neubiorev.2013.10.011. 24184743 PMC4878897

[50] S. Jung , H. Yang , B. S. Kim , K. Chu , S. K. Lee , and D. Jeon , “The Immunosuppressant Cyclosporin A Inhibits Recurrent Seizures in an Experimental Model of Temporal Lobe Epilepsy,” Neuroscience Letters 529 (2012): 133–138, 10.1016/j.neulet.2012.08.087. 22981977

[51] R. S. Zucker and W. G. Regehr , “Short‐Term Synaptic Plasticity,” Annual Review of Physiology 64 (2002): 355–405, 10.1146/annurev.physiol.64.092501.114547. 11826273

[52] Y. Bozzi , G. Provenzano , and S. Casarosa , “Neurobiological Bases of Autism–Epilepsy Comorbidity: a Focus on Excitation/Inhibition Imbalance,” European Journal of Neuroscience 47 (2018): 534–548, 10.1111/ejn.13595. 28452083

[53] R. J. Deshaies and C. A. P. Joazeiro , “RING Domain E3 Ubiquitin Ligases,” Annual Review of Biochemistry 78 (2009): 399–434, 10.1146/annurev.biochem.78.101807.093809. 19489725

[54] K. Zhou , L. Wang , Z. Sun , et al., “COP1 Acts as a Ubiquitin Ligase for PCDH9 Ubiquitination and Degradation in Human Glioma,” Molecular Neurobiology 59 (2022): 2378–2388, 10.1007/s12035-021-02634-0. 35084653

[55] S. Uljon , X. Xu , I. Durzynska , et al., “Structural Basis for Substrate Selectivity of the E3 Ligase COP1,” Structure 24 (2016): 687–696, 10.1016/j.str.2016.03.002. 27041596 PMC4856590

[56] M. Sadowski , R. Suryadinata , A. R. Tan , S. N. A. Roesley , and B. Sarcevic , “Protein Monoubiquitination and Polyubiquitination Generate Structural Diversity To Control Distinct Biological Processes,” Iubmb Life 64 (2012): 136–142, 10.1002/iub.589. 22131221

[57] G. L. Grice and J. A. Nathan , “The Recognition of Ubiquitinated Proteins by the Proteasome,” Cellular and Molecular Life Sciences 73 (2016): 3497–3506, 10.1007/s00018-016-2255-5. 27137187 PMC4980412

[58] Y. Nakamura , L. M. Darnieder , T. Z. Deeb , and S. J. Moss , “Chapter Four‐Regulation of GABA_A_Rs by Phosphorylation,” Advances in Pharmacology 72 (2015): 97–146. 25600368 10.1016/bs.apha.2014.11.008 PMC5337123

[59] T. Horie , T. Nakao , Y. Miyasaka , et al., “microRNA‐33 Maintains Adaptive Thermogenesis via Enhanced Sympathetic Nerve Activity,” Nature Communications 12 (2021): 843. 10.1038/s41467-021-21107-5 PMC7886914 33594062

[60] S. Maljevic , R. S. Møller , C. A. Reid , et al., “Spectrum of GABAA Receptor Variants in Epilepsy,” Current Opinion in Neurology 32 (2019): 183–190, 10.1097/WCO.0000000000000657. 30664068

[61] C. C. Hernandez and R. L. Macdonald , “A Structural Look at GABAA Receptor Mutations Linked to Epilepsy Syndromes,” Brain Research 1714 (2019): 234–247, 10.1016/j.brainres.2019.03.004. 30851244

[62] Y. Feng , Z.‐H. Wei , C. Liu , et al., “Genetic variations in GABA Metabolism and Epilepsy,” Seizure: European Journal of Epilepsy 101 (2022): 22–29, 10.1016/j.seizure.2022.07.007. 35850019

[63] Y.‐W. Shi , Q. Zhang , K. Cai , et al., “Synaptic Clustering Differences due to Different GABRB3 Mutations Cause Variable Epilepsy Syndromes,” Brain 142 (2019): 3028–3044, 10.1093/brain/awz250. 31435640 PMC6776116

[64] A. De Leo , A. Ugolini , X. Yu , et al., “Glucose‐Driven Histone Lactylation Promotes the Immunosuppressive Activity of Monocyte‐Derived Macrophages in Glioblastoma,” Immunity 57 (2024): 1105–1123.e8, 10.1016/j.immuni.2024.04.006. 38703775 PMC11114377

[65] G. Wang , G. Gong , X. Yang , et al., “Microglia Dld‐K127 Lactylation Promotes Parkinson's Disease via Regulating the Metabolism of Lactate‐Pyruvate Transformation,” Journal of Advanced Research (2026): S2090, 10.1016/j.jare.2026.03.016. 41812703

[66] P. Jiruska , D. Freestone , V. Gnatkovsky , and Y. Wang , “An Update on the Seizures Beget Seizures Theory,” Epilepsia 64 (2023), S13–24. 37466948 10.1111/epi.17721

[67] M. Bélanger , I. Allaman , and P. J. Magistretti , “Brain Energy Metabolism: Focus on Astrocyte‐Neuron Metabolic Cooperation,” Cell metabolism 14 (2011): p724–738. 10.1016/j.cmet.2011.08.016 22152301

[68] G. A. Dienel , “Brain Glucose Metabolism: Integration of Energetics With Function,” Physiological Reviews 99 (2019): 949–1045, 10.1152/physrev.00062.2017. 30565508

[69] Y. Su , Y. Luo , P. Zhang , et al., “Glucose‐Induced CRL4COP1‐p53 Axis Amplifies Glycometabolism to Drive Tumorigenesis,” Molecular Cell 83 (2023): 2316–2331.e7, 10.1016/j.molcel.2023.06.010. 37390815

[70] W. G. Kaelin and S. L. McKnight , “Influence of Metabolism on Epigenetics and Disease,” Cell 153 (2013): 56–69, 10.1016/j.cell.2013.03.004. 23540690 PMC3775362

[71] T. Shimazu , M. D. Hirschey , J. Newman , et al., “Suppression of Oxidative Stress by β‐Hydroxybutyrate, an Endogenous Histone Deacetylase Inhibitor,” Science 339 (2013): 211–214, 10.1126/science.1227166. 23223453 PMC3735349

[72] I. D'Andrea Meira , T. T. Romão , H. J. Pires do Prado , L. T. Krüger , M. E. P. Pires , and P. O. da Conceição , “Ketogenic Diet and Epilepsy: What We Know So Far,” Frontiers in Neuroscience 13 (2019): 5, 10.3389/fnins.2019.00005. 30760973 PMC6361831

[73] G. van Niekerk and A.‐M. Engelbrecht , “Role of PKM2 in Directing the Metabolic Fate of Glucose in Cancer: a Potential Therapeutic Target,” Cellular Oncology 41 (2018): 343–351, 10.1007/s13402-018-0383-7. 29797241 PMC12995226

[74] I. Maze , K.‐M. Noh , A. A. Soshnev , and C. D. Allis , “Every Amino Acid Matters: Essential Contributions of Histone Variants to Mammalian Development and Disease,” Nature Reviews Genetics 15 (2014): 259–271, 10.1038/nrg3673. PMC4082118 24614311

[75] Y. Fu , Z. Zhu , G. Meng , R. Zhang , and Y. Zhang , “A CRISPR‐Cas9 Based Shuffle System for Endogenous Histone H3 and H4 Combinatorial Mutagenesis,” Scientific Reports 11 (2021): 3298, 10.1038/s41598-021-82774-4. 33558622 PMC7870972

[76] R. Cai , R. Lv , X. Shi , G. Yang , and J. Jin , “CRISPR/dCas9 Tools: Epigenetic Mechanism and Application in Gene Transcriptional Regulation,” International Journal of Molecular Sciences 24 (2023): 14865, 10.3390/ijms241914865. 37834313 PMC10573330

[77] K. Hyun , J. Jeon , K. Park , and J. Kim , “Writing, Erasing and Reading Histone Lysine Methylations,” Experimental & Molecular Medicine 49 (2017): 324, 10.1038/emm.2017.11. PMC6130214 28450737

[78] H. E. Scharfman and N. J. MacLusky , “Sex Differences in the Neurobiology of Epilepsy: a Preclinical Perspective,” Neurobiology of Disease 72 (2014): 180–192. 25058745 10.1016/j.nbd.2014.07.004 PMC4252793

[79] B. L. Clossen and D. S. Reddy , “Catamenial‐Like Seizure Exacerbation in Mice With Targeted Ablation of Extrasynaptic δGABA‐a Receptors in the Brain,” Journal of Neuroscience Research 95 (2017): 1906–1916, 10.1002/jnr.24028. 28236431 PMC5561461

