# Supplementary material for: Enhanced Glycolysis‐Driven Histone H3K18 Lactylation Regulates Epileptogenesis by Modulating the E3 Ubiquitin Ligase COP1
Source: Adv Sci (Weinh). 2026 May 29;13(41):e16985. doi: 10.1002/advs.202516985 (PMC13336032; doi:10.1002/advs.202516985)
Supplement: Supplementary file 1 — Supporting File 1: advs75813‐sup‐0001‐SuppMat.docx. [file ADVS-13-e16985-s002.docx]

**
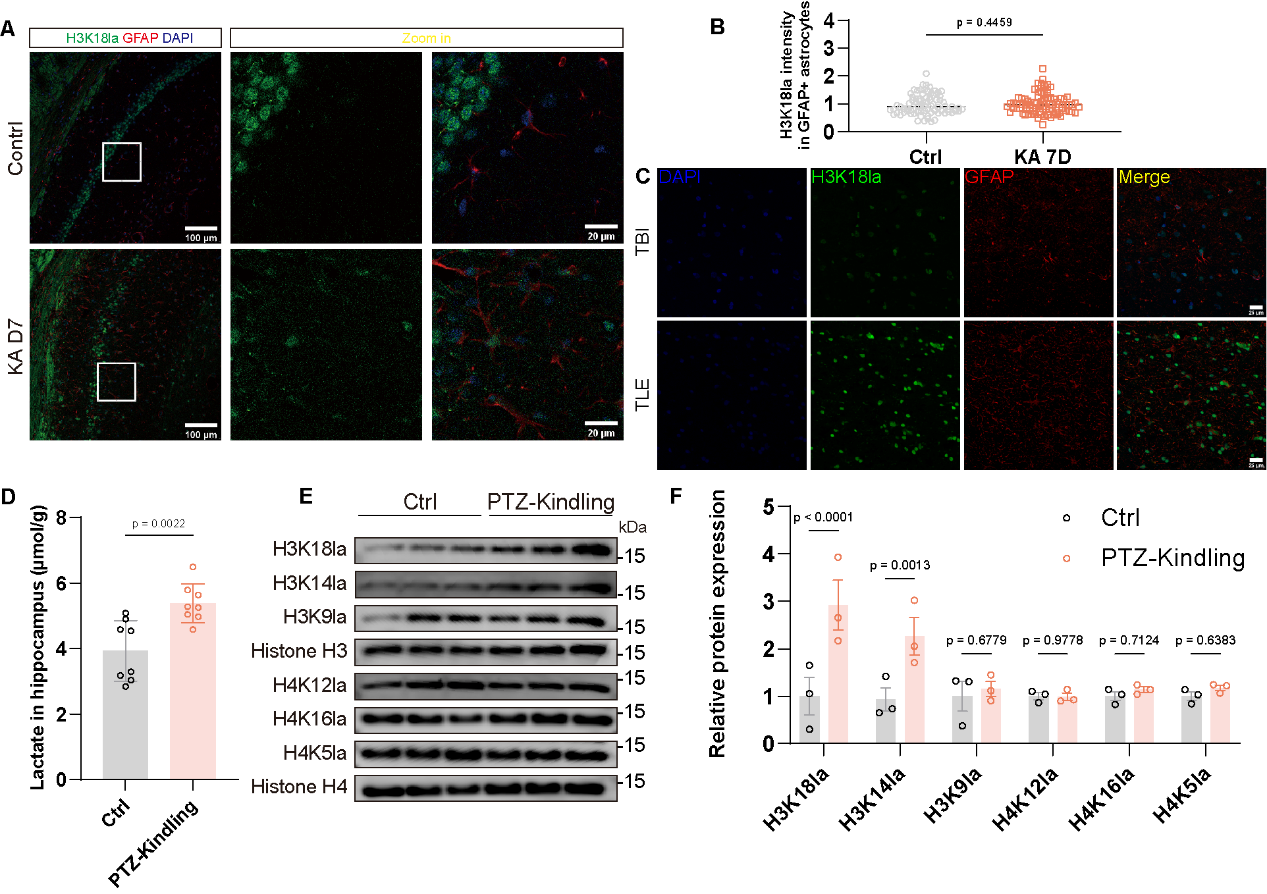
**

**Figure S1. Astrocytic H3K18la is unchanged in murine and human epileptic brain tissue, and histone lactylation is elevated in the PTZ kindling model. Related to Figure 1.**

(A) Representative confocal microscopy images of the hippocampal CA1 region from control (Ctrl) and KA-D7 mice. Sections were co-stained for H3K18la (green) and the astrocyte marker GFAP (red), with nuclei counterstained with DAPI (blue). Scale bars, 100 μm (main images) and 20 μm (insets).

(B) Quantification of H3K18la fluorescence intensity in GFAP-positive astrocytes from the mice shown in (A) (n = 3 mice per group, with 5 fields analyzed per mouse).

(C) Representative confocal microscopy images of hippocampal tissue from traumatic brain injury (TBI) controls and temporal lobe epilepsy (TLE) patients, co-stained for H3K18la (green), GFAP (red), and DAPI (blue). Scale bars, 25 μm.

(D) Quantification of lactate levels in the hippocampus of Ctrl and PTZ-kindling mice (n = 8 mice per group).

(E) Representative western blots of site-specific histone lactylation marks (H3K18la, H3K14la, H3K9la, H4K12la, H4K16la, and H4K5la) from hippocampal tissue of Ctrl and PTZ-kindling mice. Histone H3 and histone H4 were used as loading controls.

(F) Densitometric quantification of relative protein expression of histone lactylation marks shown in (E) (n = 3 mice per group).

Data are presented as mean ± SEM. Statistical significance was determined by an unpaired, two-tailed Student's t-test (B and D) or two-way ANOVA with Tukey's post hoc test (F). Exact p values are indicated in the figure.


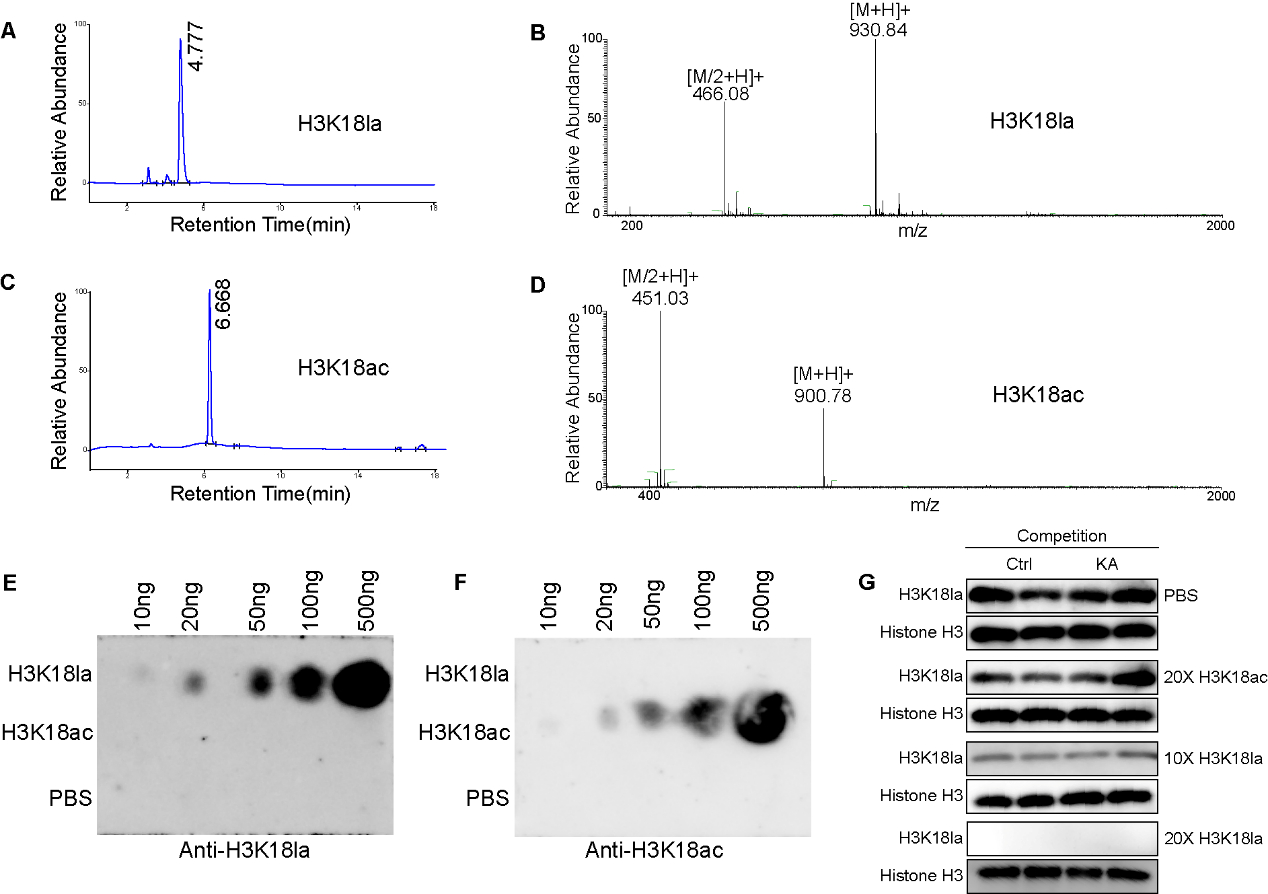


**Figure S2. Mass spectrometric confirmation and antibody specificity validation of the Anti-H3K18la antibody. Related to Figure 1.**

(A) Extracted ion chromatogram from HPLC/MS/MS analysis of the synthetic H3K18la peptide.

(B) MS/MS spectrum of the synthetic H3K18la peptide.

(C) Extracted ion chromatogram from HPLC/MS/MS analysis of the synthetic H3K18ac peptide.

(D) MS/MS spectrum of the synthetic H3K18ac peptide.

(E) Dot blot performed with the Anti-H3K18la antibody. Dots contain 10, 20, 50, 100, or 500 ng of H3K18la-modified, H3K18ac-modified, or unmodified (PBS) peptide.

(F) Dot blot performed with the Anti-H3K18ac antibody using the same peptide panel as in (E).

(G) Peptide competition assay. The Anti-H3K18la antibody was pre-incubated with PBS, a 20-fold excess of H3K18ac peptide, or a 10- or 20-fold excess of H3K18la peptide prior to immunoblotting of hippocampal lysates from Ctrl and KA-treated mice. Histone H3 was used as a loading control.


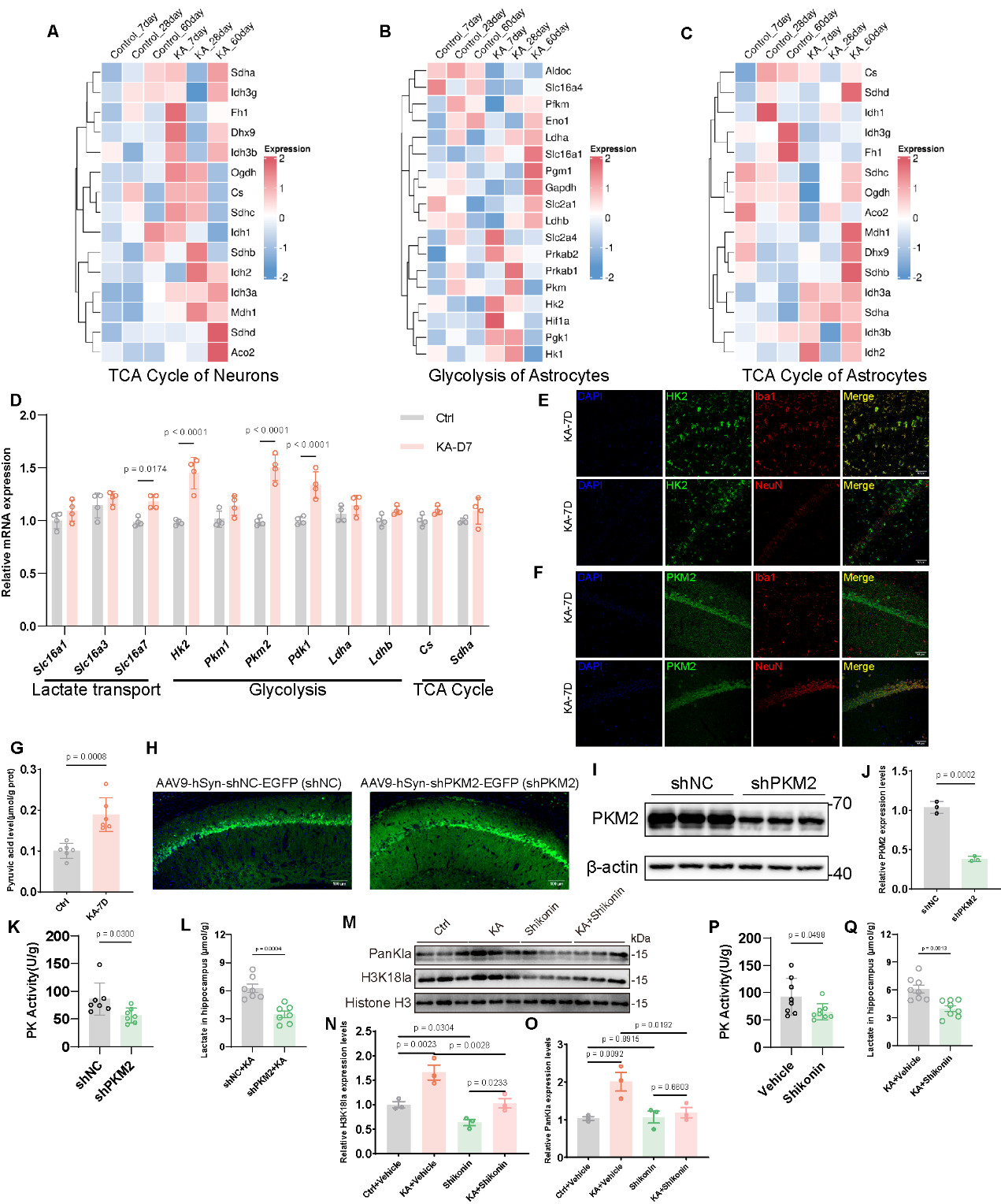


**Figure S3. Cell-type-specific metabolic reprogramming and functional validation of PKM2 targeting in the epileptic hippocampus. Related to Figure 2.**

(A–C) Heatmaps from snRNA-seq analysis showing z-score normalized expression of TCA cycle genes in neurons (A), and glycolysis (B) and TCA cycle (C) genes in astrocytes, across control and KA-treated mice at 7, 28, and 60 days post-injection.

(D) qRT-PCR quantification of mRNA expression for lactate transport (*Slc16a1, Slc16a3, Slc16a7*), glycolysis (*Hk2, Pkm1, Pkm2, Pdk1, Ldha, Ldhb*), and TCA cycle (*Cs, Sdha*) genes in hippocampal tissue from control and KA-D7 mice (n = 4 mice per group). Exact p values for significant comparisons are as follows: *Hk2*, p = 0.0174; *Pkm2*, p < 0.0001; *Pdk1*, p < 0.0001; *Ldha*, p < 0.0001. All other genes were not significantly different (p > 0.05).

(E and F) Representative confocal immunofluorescence images of hippocampal sections from KA-D7 mice, co-stained for HK2 (green) with Iba1 or NeuN (red) (E), and PKM2 (green) with Iba1 or NeuN (red) (F). Nuclei were counterstained with DAPI (blue). Scale bars, 50 μm.

(G) Pyruvic acid levels in hippocampal lysates from control and KA-D7 mice (n = 6 mice per group).

(H) Representative fluorescence images showing EGFP expression in hippocampal neurons following injection of AAV9-hSyn-shNC-EGFP or AAV9-hSyn-shPKM2-EGFP. Scale bar, 100 μm.

(I and J) Representative Western blot (I) and quantification (J) of PKM2 protein levels confirming knockdown efficiency in shNC and shPKM2 mice. β-Actin was used as a loading control (n = 3 mice per group).

(K and L) PK enzymatic activity (K) and lactate levels (L) in hippocampal lysates from shNC+KA and shPKM2+KA mice (n = 7 mice per group).

(M–O) Representative Western blots (M) and quantification of H3K18la (N) and PanKla (O) in hippocampal lysates from Ctrl+Vehicle, KA+Vehicle, Shikonin, and KA+Shikonin groups. Histone H3 was used as a loading control (n = 3 mice per group).

(P and Q) PK enzymatic activity (P) and lactate levels (Q) in hippocampal lysates from vehicle- and shikonin-treated mice (n = 8 and 7 mice per group, respectively).

Data are presented as mean ± SEM. Statistical significance was determined by an unpaired, two-tailed Student's t-test (G, J, K, L, and P) or one-way ANOVA with Tukey's post hoc test (N, O, and Q). Exact p values are indicated in the figure unless otherwise stated above.


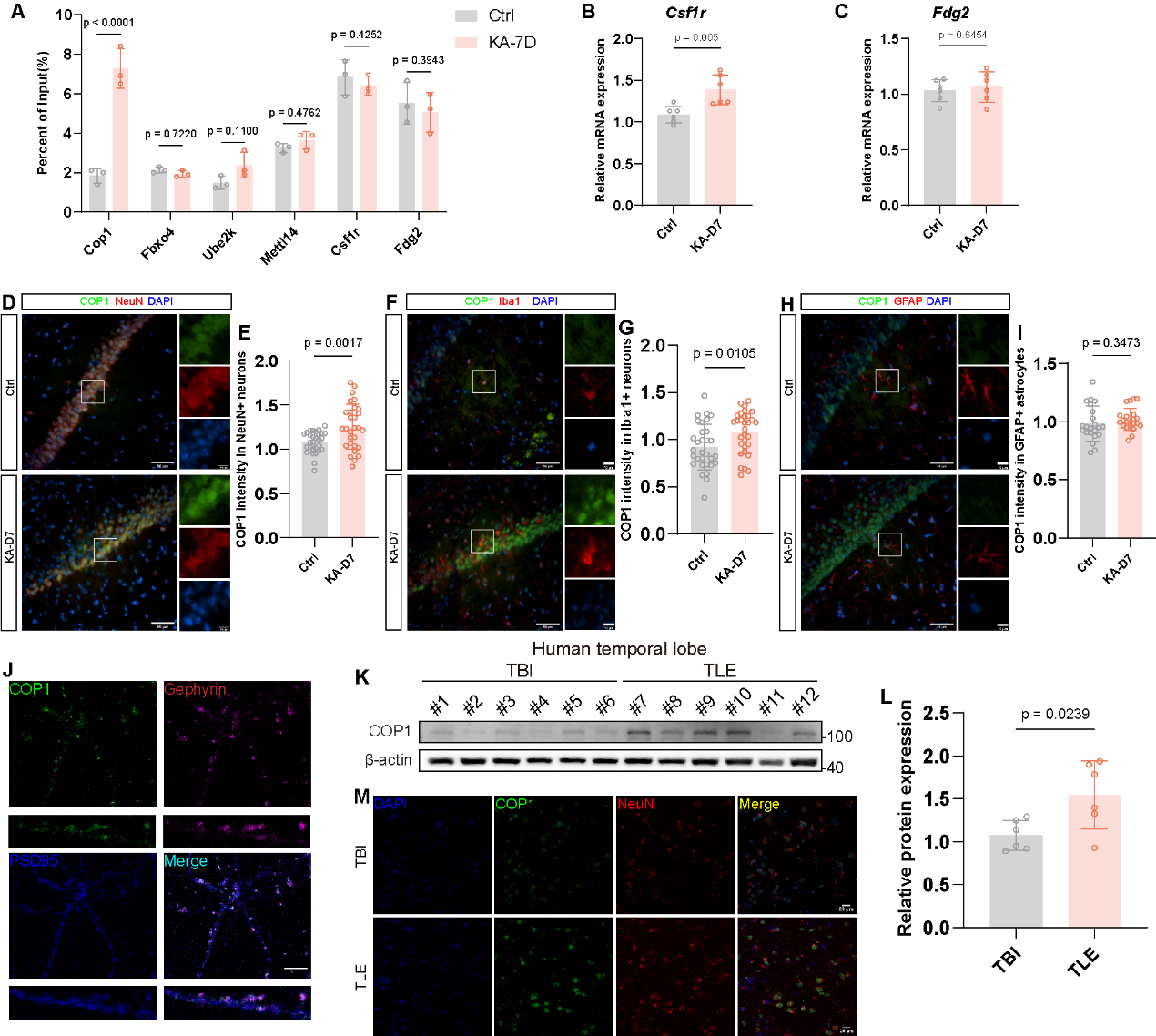


**Figure S4. COP1 is upregulated in neurons and of the epileptic hippocampus, colocalizes with inhibitory postsynaptic scaffolds in primary neurons, and is elevated in human epileptic tissue. Related to Figure 3.**

(A) ChIP-qPCR analysis of H3K18la enrichment at the promoters of additional ubiquitin-proteasome-related target genes (*Fbxo4, Ube2k, Mettl14, Csf1r, and Fdg2*) in control and KA-D7 hippocampal tissue, expressed as percentage of input (n = 3 mice per group).

(B and C) qRT-PCR quantification of *Csf1r* (B) and *Fdg2* (C) mRNA expression in hippocampal tissue from control and KA-D7 mice (n = 6 mice per group).

(D and E) Representative confocal images of hippocampal sections from control and KA-D7 mice co-stained for COP1 (green), NeuN (red), and DAPI (blue) (D). Quantification of COP1 fluorescence intensity in NeuN-positive neurons (E) (n = 3 mice, 5 fields per mouse, 40–60 cells per field). Scale bars, 50 μm (main images) and 10 μm (insets).

(F and G) Representative confocal images co-stained for COP1 (green), Iba1 (red), and DAPI (blue) (F), and quantification of COP1 fluorescence intensity in Iba1-positive microglia (G) (n = 3 mice, 5 fields per mouse, 40–60 cells per field). Scale bars as in (D).

(H and I) Representative confocal images co-stained for COP1 (green), GFAP (red), and DAPI (blue) (H), and quantification of COP1 fluorescence intensity in GFAP-positive astrocytes (I) (n = 3 mice, 5 fields per mouse, 40–60 cells per field). Scale bars as in (D).

(J) Representative confocal images of primary hippocampal neurons (DIV14) co-stained for COP1 (green), Gephyrin (magenta), and PSD95 (blue), showing colocalization of COP1 with the inhibitory postsynaptic scaffold Gephyrin. Scale bar, 20 μm.

(K and L) Representative Western blots (K) and quantification (L) of COP1 protein levels in temporal lobe tissue from TBI controls and TLE patients. β-Actin was used as a loading control (n = 6 patients per group).

(M) Representative confocal images of hippocampal tissue from TBI and TLE patients co-stained for COP1 (green), NeuN (red), and DAPI (blue). Scale bar, 25 μm.

Data are presented as mean ± SEM. Statistical significance was determined by an unpaired, two-tailed Student's t-test (B, C, E, G, I, and L). Exact p values are indicated in the figure.


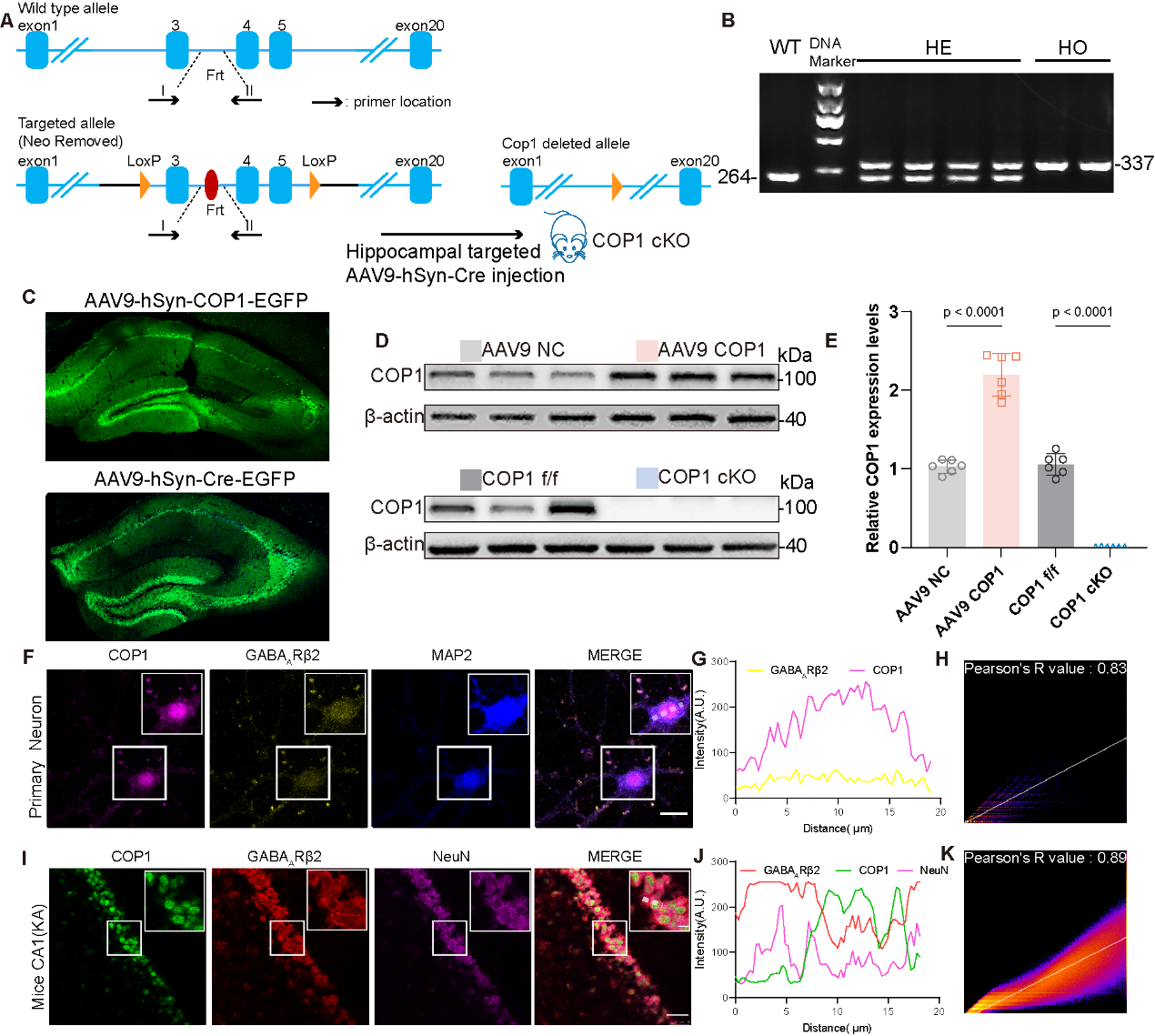


**Figure S5. Validation of neuronal COP1 manipulation and its colocalization with GABA_A_Rβ2, Related to Figure 4.**

(A) Schematic of the conditional *Cop1* knockout strategy. LoxP sites were inserted flanking exons 3–5 of the *Cop1* locus, and hippocampal-specific knockout was achieved by AAV9-hSyn-Cre injection.

(B) Representative PCR genotyping gel showing wild-type (WT), heterozygous (HE), and homozygous (HO) alleles. Expected band sizes: 264 bp (deleted) and 337 bp (floxed).

(C) Virus infection efficiency of AAV9-hSyn-COP1-EGFP (for overexpression) or AAV9-hSyn-Cre-EGFP (for conditional knockout) into the CA1 region of the hippocampus.

(D and E) Representative Western blots (D) and quantification (E) of COP1 protein levels in AAV9-NC, AAV9-COP1, COP1 f/f, and COP1 cKO hippocampal tissue, confirming overexpression and conditional knockout efficiency. β-Actin was used as a loading control (n = 6 mice per group).

(F–H) Colocalization of COP1 and GABA_A_Rβ2 in primary hippocampal neurons. (F) Representative confocal images co-stained for COP1 (magenta), GABA_A_Rβ2 (yellow), and MAP2 (blue). Scale bar, 5 μm. (G) Line-scan fluorescence intensity profiles of COP1 and GABA_A_Rβ2 along the region indicated in (F). (H) Pearson's correlation coefficient for COP1/GABA_A_Rβ2 colocalization (R = 0.83).

(I–K) Colocalization of COP1 and GABA_A_Rβ2 in CA1 hippocampal neurons from KA-treated mice. (I) Representative confocal images co-stained for COP1 (green), GABAARβ2 (red), and NeuN (magenta). Scale bar, 20 μm. (J) Line-scan fluorescence intensity profiles of COP1, GABA_A_Rβ2, and NeuN. (K) Pearson's correlation coefficient for COP1/GABA_A_Rβ2 colocalization (R = 0.89).

Data are presented as mean ± SEM. Statistical significance was determined by one-way ANOVA with Tukey's post hoc test (E). Exact p values are indicated in the figure.


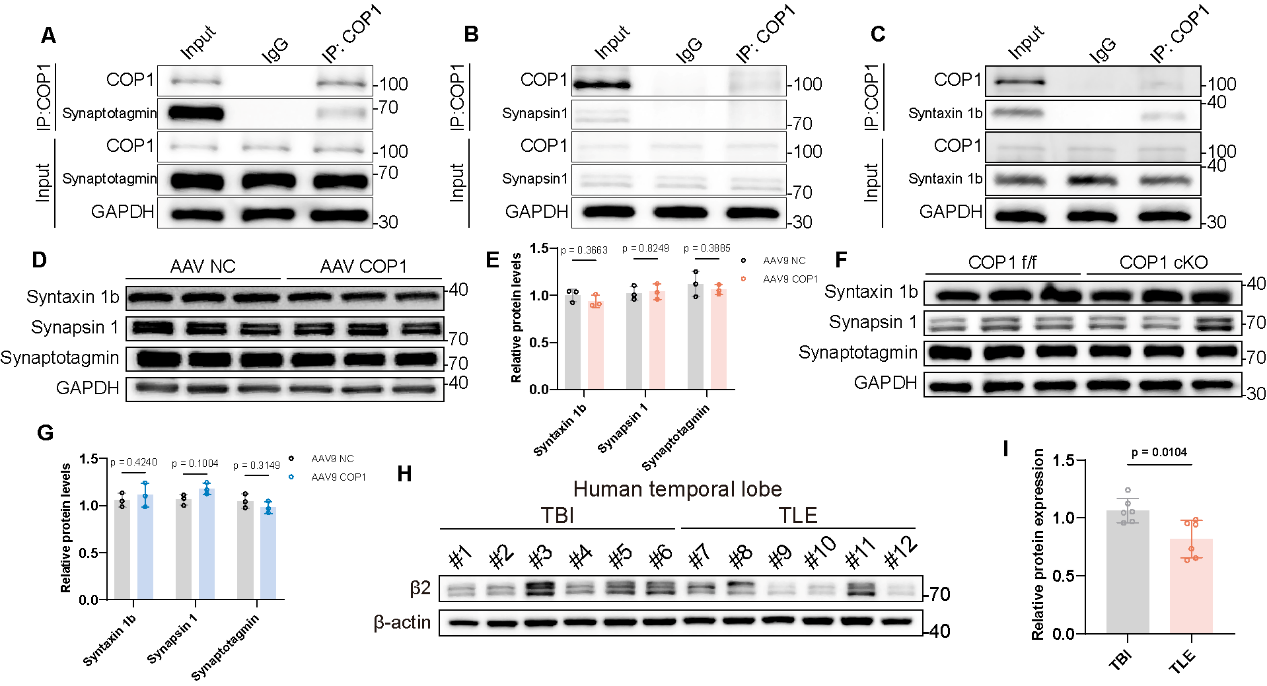


**Figure S6. COP1 selectively targets GABAARβ2, but not other synaptic proteins, for degradation, and GABAARβ2 is downregulated in human epileptic tissue. Related to Figure 5.**

(A–C) Co-immunoprecipitation using anti-COP1 antibody in KA-induced epileptic hippocampal lysates, with GAPDH detected in input lanes as a loading control. Immunoblotting of Synaptotagmin (A), Synapsin 1 (B), and Syntaxin 1b (C).

(D and E) Representative Western blots (D) and quantification (E) of Syntaxin 1b, Synapsin 1, and Synaptotagmin protein levels in hippocampal lysates from AAV9-NC and AAV9-COP1 mice. GAPDH was used as a loading control (n = 3 mice per group).

(F and G) Representative Western blots (F) and quantification (G) of Syntaxin 1b, Synapsin 1, and Synaptotagmin protein levels in hippocampal lysates from COP1 f/f and COP1 cKO mice. GAPDH was used as a loading control (n = 3 mice per group).

(H and I) Representative Western blots (H) and quantification (I) of GABA_A_Rβ2 protein levels in temporal lobe tissue from TBI controls and TLE patients (#1–6, TBI; #7–12, TLE). β-Actin was used as a loading control (n = 6 patients per group).

Data are presented as mean ± SEM. Statistical significance was determined by an unpaired, two-tailed Student's t-test (E, G, and I). Exact p values are indicated in the figure.
