# Supplementary material for: Enhanced Glycolysis‐Driven Histone H3K18 Lactylation Regulates Epileptogenesis by Modulating the E3 Ubiquitin Ligase COP1
Source: Adv Sci (Weinh). 2026 May 29;13(41):e16985. doi: 10.1002/advs.202516985 (PMC13336032; doi:10.1002/advs.202516985)
Supplement: Supplementary file 2 — Supporting File 2: advs75813‐sup‐0002‐TableS1‐S3.zip. [file ADVS-13-e16985-s001.zip › TableS1.docx]

Table S1.1 qRT-PCR primer sequences

| **Gene** | **Primer (5’→3’)** |
| --- | --- |
| *Pkm1* (Forward) | CTGGGGAAGTGGCTGAAAGATG |
| *Pkm1* (Reverse) | GCCAGACTCCGTCAGAACTCGT |
| *Pkm2* (Forward) | ATTATTTGAGGAACTCCGCCGC |
| *Pkm2* (Reverse) | GCCAGACTCCGTCAGAACTCGT |
| *Hk2* (Forward) | TGATCGCCTGCTTATTCACGG |
| *Hk2* (Reverse) | AACCGCCTAGAAATCTCCAGA |
| *Pdk1* (Forward) | GGACTTCGGGTCAGTGAATGC |
| *Pdk1* (Reverse) | TCCTGAGAAGATTGTCGGGGA |
| *Slc16a1* (Forward) | GCTGGCTGTCATGTATGGTG |
| *Slc16a1* (Reverse) | CCCAGTAGCTCCGATGATGA |
| *Slc16a3* (Forward) | CTACATCAGCCGCAAGAAGG |
| *Slc16a3* (Reverse) | CTCCGAGGAGTTCCATGAGG |
| *Cop1* (Forward) | TGGCAAAGAGCAGTTCCTGAAG |
| *Cop1* (Reverse) | AGCATCAGTCCGTAGTCTTGGT |
| *Gabrb2* (Forward) | GCCTACAGCATCATCAGTGGAC |
| *Gabrb2* (Reverse) | TGAACACCAGCAGCAACAGAGT |
| *Csf1r* (Forward) | TGCCTTCTCCTTCACCTTCAGC |
| *Csf1r* (Reverse) | GGCAGAGTGAAGACCAGGATGT |
| *Fgd2* (Forward) | ACAGCCAAGATGCTGTCCTACA |
| *Fgd2* (Reverse) | TGATGGTCTTGATGACCTCGGT |
| *Actb* (Forward) | GGCTGTATTCCCCTCCATCG |
| *Actb* (Reverse) | CCAGTTGGTAACAATGCCATGT |

**Table S1.2 ChIP-qPCR primer sequences**

| **Gene** | **Site** | **Primer (5’→3’)** |
| --- | --- | --- |
| *Cop1* | Site A | F: AGGCTCAGTGCTTCAAGACC R: TGGAGGTCCAGGTTCACTCA |
| *Cop1* | Site B | F: GCACCTGTAATCCCAGCACT R: CTGCCTCAGCCTCCCAAGTA |
| *Cop1* | Site C | F: CTGAGGTCAGGAGTTCGAGA R: GCAGAAGCCACGTTCTACCT |
| *Cop1* | Site D | F: AGCCGAGATCACGCCACTAC R: CAGCCAGGCTGGTCTCAAAC |
