# Supplementary material for: Enhanced Glycolysis‐Driven Histone H3K18 Lactylation Regulates Epileptogenesis by Modulating the E3 Ubiquitin Ligase COP1
Source: Adv Sci (Weinh). 2026 May 29;13(41):e16985. doi: 10.1002/advs.202516985 (PMC13336032; doi:10.1002/advs.202516985)
Supplement: Supplementary file 2 — Supporting File 2: advs75813‐sup‐0002‐TableS1‐S3.zip. [file ADVS-13-e16985-s001.zip › TableS2.docx]

Table S2. Clinical characteristics of TLE patients and TBI patients

| **Cases** | **Gender**  **(M/F)** | **Age**  **(years)** | **AEDs before surgery** | **Resection tissue** | **Neuropathological**  **diagnosis** |
| --- | --- | --- | --- | --- | --- |
| TLE 1 | F | 18 | VPA, CBZ, CZP | TN, L | NL, G |
| TLE 2 | F | 36 | VPA, CBZ, TPM | TN, R | NL, G |
| TLE 3 | M | 21 | VPA, CBZ, TPM | TN, L | NL, G |
| TLE 4 | F | 12 | VPA, CBZ, LTG | TN, L | NL, G |
| TLE 5 | F | 13 | VPA, CBZ, LTG | TN, R | NL, G |
| TLE 6 | M | 26 | VPA, CBZ, PHT | TN, L | NL, G |
| TBI 1 | M | 25 | None | TN, L | N |
| TBI 2 | M | 21 | None | TN, L | N |
| TBI 3 | F | 29 | None | TN, R | N |
| TBI 4 | M | 18 | None | TN, R | N |
| TBI 5 | F | 33 | None | TN, L | N |
| TBI 6 | M | 38 | None | TN, R | N |

CBZ, carbamazepine; CZP, clonazepam; F, female; G, gliosis; L, left; LTG, lamotrigine; M, male; N, relative normal; NL, neuron loss; PHT, phenytoin; R, right; TN, temporal neocortex; TPM, topiramate; VPA, valproate.
