# Supplementary material for: Enhanced Glycolysis‐Driven Histone H3K18 Lactylation Regulates Epileptogenesis by Modulating the E3 Ubiquitin Ligase COP1
Source: Adv Sci (Weinh). 2026 May 29;13(41):e16985. doi: 10.1002/advs.202516985 (PMC13336032; doi:10.1002/advs.202516985)
Supplement: Supplementary file 2 — Supporting File 2: advs75813‐sup‐0002‐TableS1‐S3.zip. [file ADVS-13-e16985-s001.zip › TableS3.docx]

| Abbreviation | Full term |
| --- | --- |
| AAV | adeno-associated virus |
| ACSF | artificial cerebrospinal fluid |
| AMPA | α-amino-3-hydroxy-5-methyl-4-isoxazolepropionic acid |
| ChIP-seq | chromatin immunoprecipitation sequencing |
| CHX | cycloheximide |
| cKO | conditional knockout |
| Co-IP | co-immunoprecipitation |
| COP1 | constitutively photomorphogenic 1 |
| DAPI | 4′,6-diamidino-2-phenylindole |
| DEG | differentially expressed gene |
| DIV | days in vitro |
| DMSO | dimethyl sulfoxide |
| DNQX | 6,7-dinitroquinoxaline-2,3-dione |
| D-APV | D-(–)-2-amino-5-phosphonopentanoic acid |
| E/I | excitation/inhibition |
| EEG | electroencephalogram |
| FDR | false discovery rate |
| FPKM | fragments per kilobase of transcript per million mapped reads |
| GABA | γ-aminobutyric acid |
| GABA_A_Rβ2 | GABAA receptor β2 subunit |
| GEO | Gene Expression Omnibus |
| GFAP | glial fibrillary acidic protein |
| GLUT1 | glucose transporter 1 |
| GO | Gene Ontology |
| GSEA | gene set enrichment analysis |
| H3K18ac | histone H3 lysine 18 acetylation |
| HDAC | histone deacetylase |
| HK2 | hexokinase 2 |
| i.p. | intraperitoneal |
| Iba1 | ionized calcium-binding adaptor molecule 1 |
| IF | immunofluorescence |
| IGV | Integrative Genomics Viewer |
| IP-MS | immunoprecipitation-mass spectrometry |
| KA | kainic acid |
| KD | knockdown |
| KEGG | Kyoto Encyclopedia of Genes and Genomes |
| LDH | lactate dehydrogenase |
| MCT2 | monocarboxylate transporter 2 |
| mIPSC | miniature inhibitory postsynaptic current |
| NaLa | sodium lactate |
| NC | negative control |
| NES | normalized enrichment score |
| NMDA | N-methyl-D-aspartate |
| PanKla | pan-lysine lactylation |
| PKM2 | pyruvate kinase M2 |
| PM | plasma membrane |
| PPR | paired-pulse ratio |
| PTZ | pentylenetetrazol |
| RNA-seq | RNA sequencing |
| RT-qPCR | reverse transcription-quantitative polymerase chain reaction |
| SE | status epilepticus |
| sEPSC | spontaneous excitatory postsynaptic current |
| sIPSC | spontaneous inhibitory postsynaptic current |
| snRNA-seq | single nucleus RNA sequencing |
| SRS | spontaneous recurrent seizures |
| STRING | Search Tool for the Retrieval of Interacting Genes/Proteins |
| TBI | traumatic brain injury |
| TLE | temporal lobe epilepsy |
| TSS | transcription start site |
| TTX | tetrodotoxin |
| UMAP | Uniform Manifold Approximation and Projection |
| UPS | ubiquitin-proteasome system |
| WT | wild-type |
