# Supplementary figures and images for: Enhanced Glycolysis‐Driven Histone H3K18 Lactylation Regulates Epileptogenesis by Modulating the E3 Ubiquitin Ligase COP1
Source: Adv Sci (Weinh). 2026 May 29;13(41):e16985. doi: 10.1002/advs.202516985 (PMC13336032; doi:10.1002/advs.202516985)

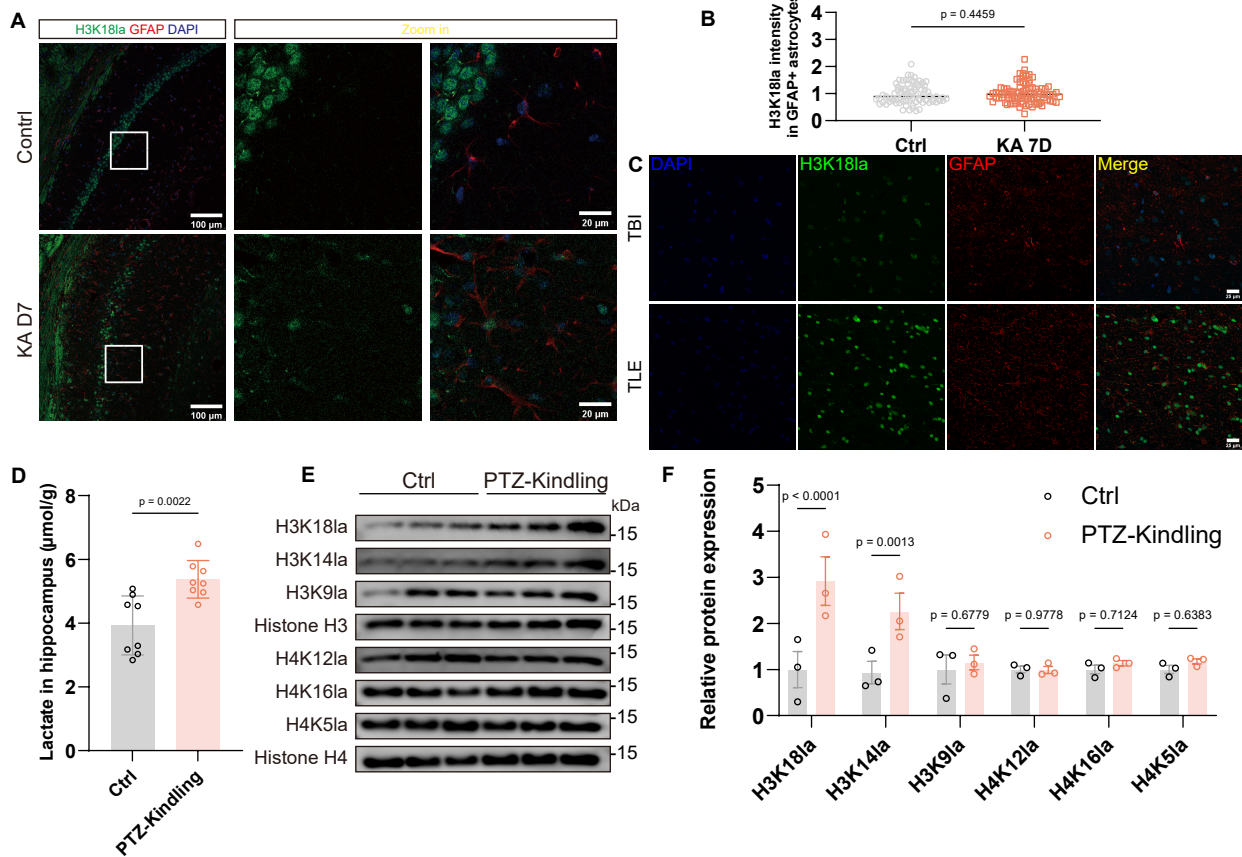

Supplement: Supplementary file 3 — Supporting File 3: advs75813‐sup‐0003‐Figures.zip. [file ADVS-13-e16985-s003.zip › SF1-RelatedToFig1.pdf]

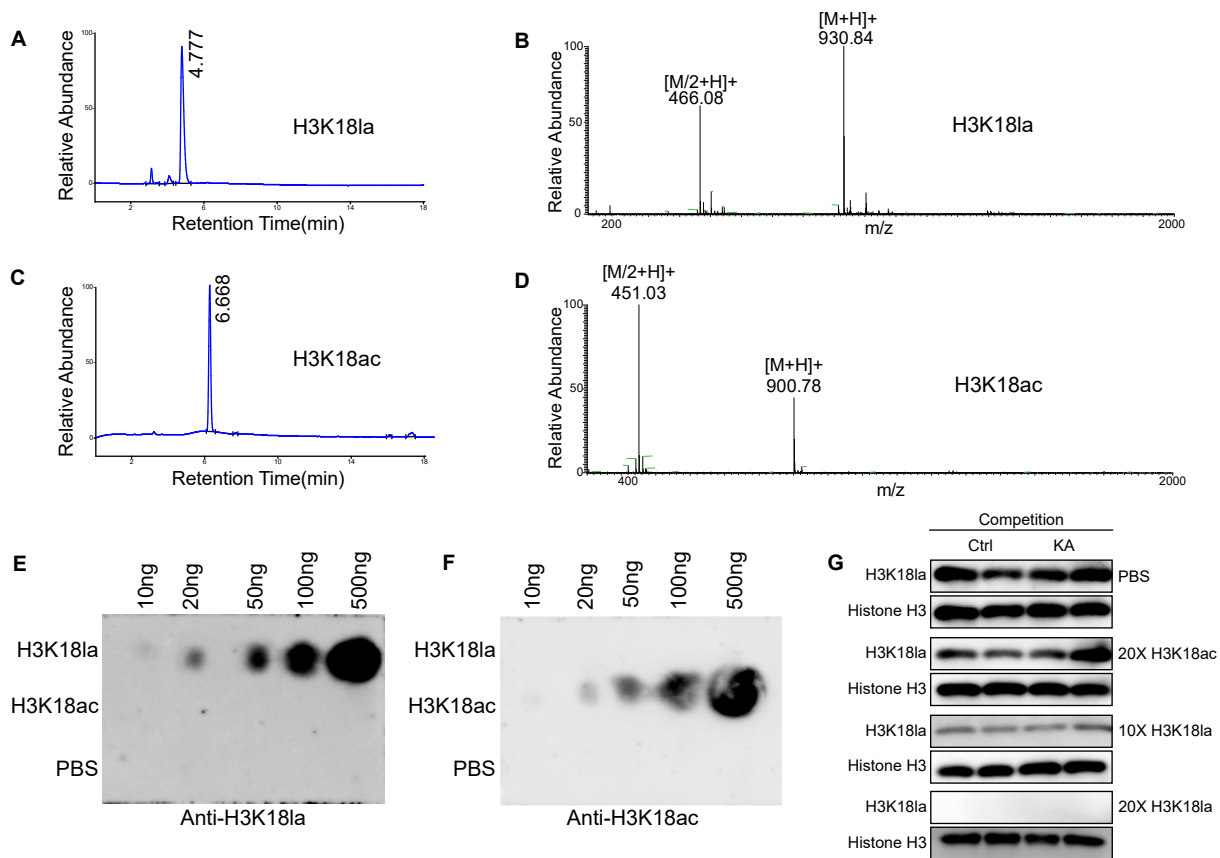

Supplement: Supplementary file 3 — Supporting File 3: advs75813‐sup‐0003‐Figures.zip. [file ADVS-13-e16985-s003.zip › SF2-RelatedToFig1.pdf]

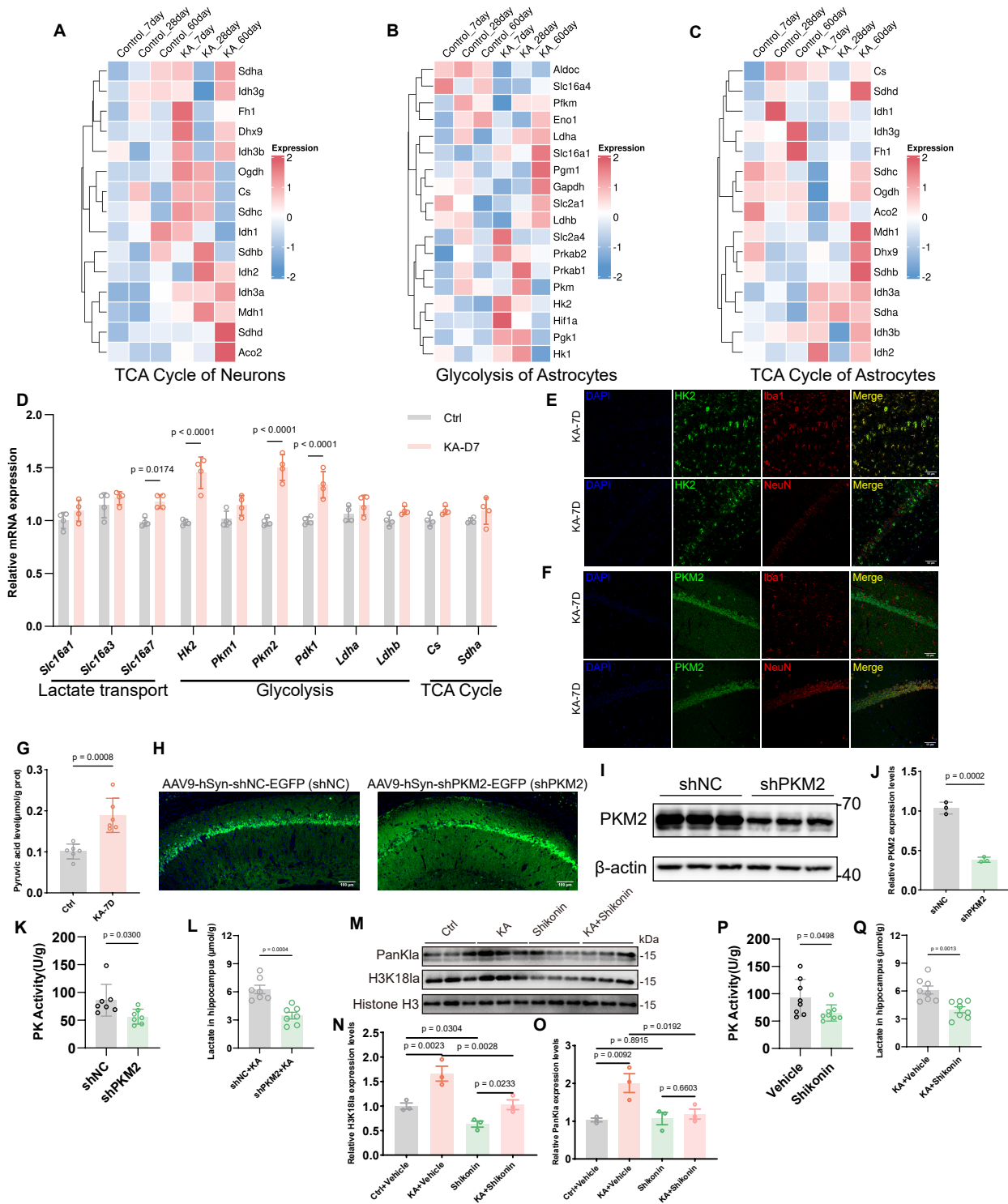

Supplement: Supplementary file 3 — Supporting File 3: advs75813‐sup‐0003‐Figures.zip. [file ADVS-13-e16985-s003.zip › SF3-RelatedToFig2.pdf]

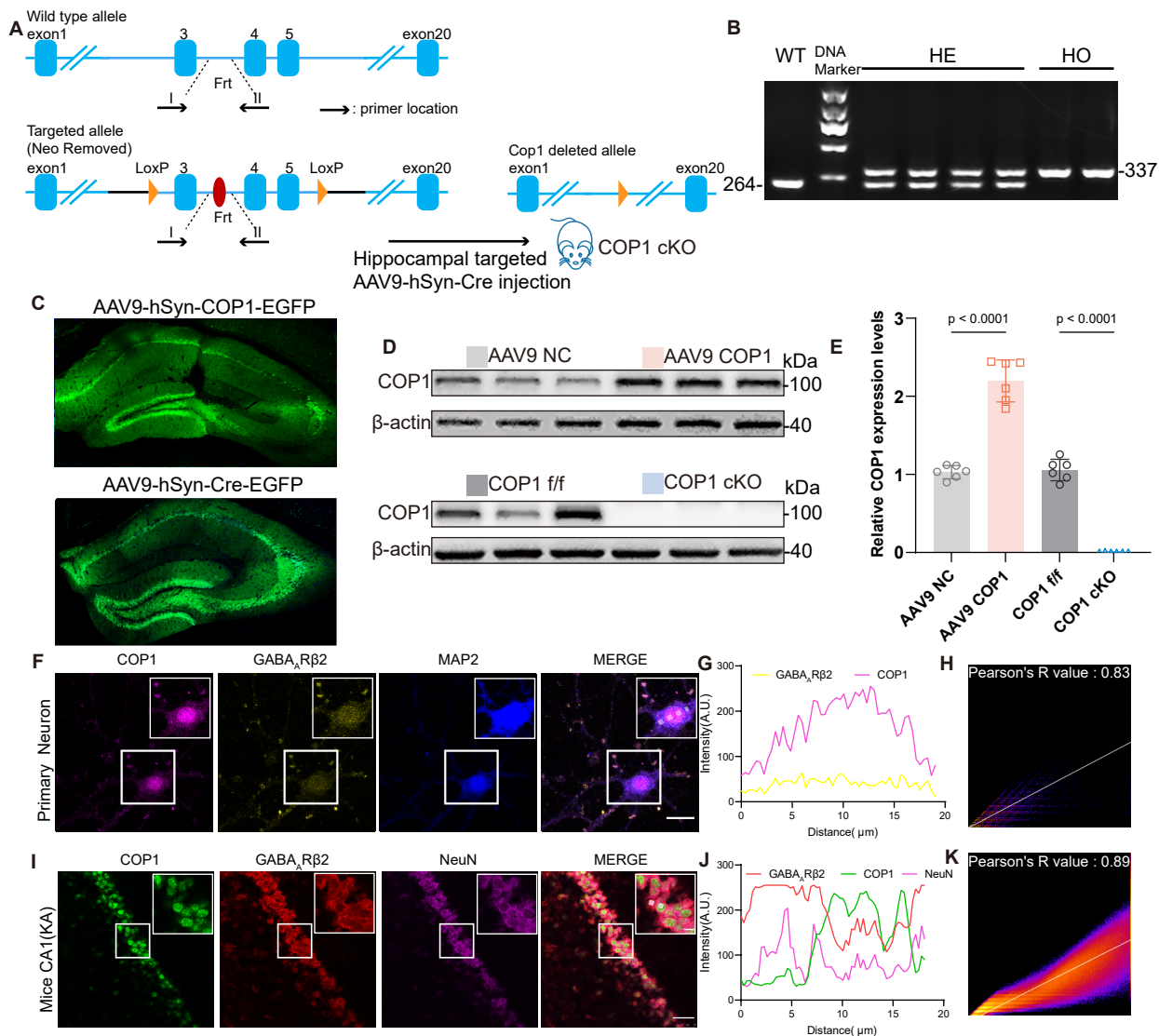

Supplement: Supplementary file 3 — Supporting File 3: advs75813‐sup‐0003‐Figures.zip. [file ADVS-13-e16985-s003.zip › SF5-RelatedToFig4.pdf]

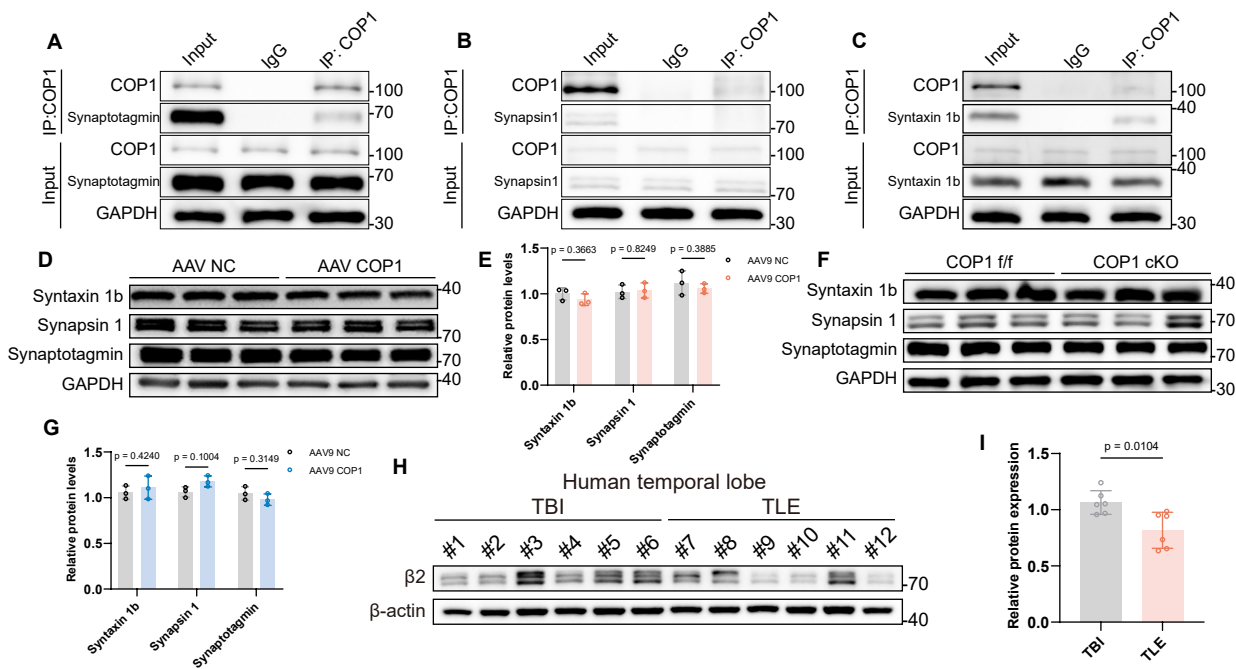

Supplement: Supplementary file 3 — Supporting File 3: advs75813‐sup‐0003‐Figures.zip. [file ADVS-13-e16985-s003.zip › SF6-RelatedToFig5.pdf]
